# Genome-Wide Identification of Essential and Auxiliary Gene Sets for Magnetosome Biosynthesis in Magnetospirillum gryphiswaldense

**DOI:** 10.1128/mSystems.00565-20

**Published:** 2020-11-17

**Authors:** Karen T. Silva, Margarete Schüler, Frank Mickoleit, Theresa Zwiener, Frank D. Müller, Ram Prasad Awal, Alfons Weig, Andreas Brachmann, René Uebe, Dirk Schüler

**Affiliations:** a Department of Microbiology, University of Bayreuth, Bayreuth, Germany; b KeyLab Genome Analysis and Bioinformatics, University of Bayreuth, Bayreuth, Germany; c Genetics Section, Bio Center, Ludwig-Maximilians-Universität München, Munich, Germany; Institute of Soil Science, Chinese Academy of Sciences

**Keywords:** magnetosome biosynthesis, microbial genetics, transposon mutagenesis

## Abstract

Magnetotactic bacteria (MTB) stand out by their ability to manufacture membrane-enclosed magnetic organelles, so-called magnetosomes. Previously, it has been assumed that a genomic region of approximately 100 kbp, the magnetosome island (MAI), harbors all genetic determinants required for this intricate biosynthesis process. Recent evidence, however, argues for the involvement of additional auxiliary genes that have not been identified yet. In the present study, we set out to delineate the full gene complement required for magnetosome production in the alphaproteobacterium Magnetospirillum gryphiswaldense using a systematic genome-wide transposon mutagenesis approach. By an optimized procedure, a Tn*5* insertion library of 80,000 clones was generated and screened, yielding close to 200 insertants with mild to severe impairment of magnetosome biosynthesis. Approximately 50% of all Tn*5* insertion sites mapped within the MAI, mostly leading to a nonmagnetic phenotype. In contrast, in the majority of weakly magnetic Tn*5* insertion mutants, genes outside the MAI were affected, which typically caused lower numbers of magnetite crystals with partly aberrant morphology, occasionally combined with deviant intracellular localization. While some of the Tn*5*-struck genes outside the MAI belong to pathways that have been linked to magnetosome formation before (e.g., aerobic and anaerobic respiration), the majority of affected genes are involved in so far unsuspected cellular processes, such as sulfate assimilation, oxidative protein folding, and cytochrome *c* maturation, or are altogether of unknown function. We also found that signal transduction and redox functions are enriched in the set of Tn*5* hits outside the MAI, suggesting that such processes are particularly important in support of magnetosome biosynthesis.

**IMPORTANCE** Magnetospirillum gryphiswaldense is one of the few tractable model magnetotactic bacteria (MTB) for studying magnetosome biomineralization. So far, knowledge on the genetic determinants of this complex process has been mainly gathered using reverse genetics and candidate approaches. In contrast, nontargeted forward genetics studies are lacking, since application of such techniques in MTB has been complicated for a number of technical reasons. Here, we report on the first comprehensive transposon mutagenesis study in MTB, aiming at systematic identification of auxiliary genes necessary to support magnetosome formation in addition to key genes harbored in the magnetosome island (MAI). Our work considerably extends the candidate set of novel subsidiary determinants and shows that the full gene complement underlying magnetosome biosynthesis is larger than assumed. In particular, we were able to define certain cellular pathways as specifically important for magnetosome formation that have not been implicated in this process so far.

## Abstract (precis)

Magnetospirillum gryphiswaldense is one of the few tractable model magnetotactic bacteria (MTB) for studying magnetosome biomineralization. So far, knowledge on the genetic determinants of this complex process has been mainly gathered using reverse genetics and candidate approaches. In contrast, nontargeted forward genetics studies are lacking, since application of such techniques in MTB has been complicated for a number of technical reasons. Here, we report on the first comprehensive transposon mutagenesis study in MTB, aiming at systematic identification of auxiliary genes necessary to support magnetosome formation in addition to key genes harbored in the magnetosome island (MAI). Our work considerably extends the candidate set of novel subsidiary determinants and shows that the full gene complement underlying magnetosome biosynthesis is larger than assumed. In particular, we were able to define certain cellular pathways as specifically important for magnetosome formation that have not been implicated in this process so far.

## INTRODUCTION

Magnetotactic bacteria (MTB) are able to navigate in the geomagnetic field by virtue of unique intracellular organelles, so-called magnetosomes. These magnetic field sensors are membrane enclosed crystals of a magnetic iron mineral, magnetite (Fe_3_O_4_) or greigite (Fe_3_S_4_), which are aligned in one or more intracellular chains by specific cytoskeletal structures. From this ordered crystal arrangement, a magnetic moment results which orients the bacterial cell along geomagnetic field lines (1–4). Impressive progress has been made during the last 2 decades in unraveling the genetic circuitry behind magnetosome formation, mostly through the study of two model organisms, the *Alphaproteobacteria* Magnetospirillum gryphiswaldense MSR-1 (5, 6) and Magnetospirillum magnetotacticum AMB-1 (7). This revealed that the biosynthesis of magnetosomes (magbiosyn) in bacterial cells is an intricate stepwise process which comprises the (i) invagination of the cytoplasmic membrane to form the magnetosome membrane (MM), either as vesicle-like permanent invagination or as detached vesicle, (ii) sorting of magnetosome proteins to the MM, either prior to, concomitantly with, or after invagination, (iii) iron transport into the vesicle and mineralization as magnetic crystal, and (iv) magnetosome chain assembly and cellular positioning for segregation during cell division (8–11). The multitude of intertwined actions underlying these stages is orchestrated and tightly controlled by more than 30 genes located in a dedicated genomic region called magnetosome island (MAI) (12, 13). It harbors all so far known specific magbiosyn determinants, which are organized in five characterized gene clusters/operons (*feoAB1*, *mms6*, *mamGFDC*, *mamXY*, and *mamAB*) (10) that were first identified by a reverse genetics approach based on a combination of proteomics and comparative genomics (14). These key gene clusters of the MAI are separated by stretches harboring genes of yet unknown but irrelevant function for magnetosome biosynthesis (15, 16). For M. gryphiswaldense, it has been shown that the largest of these potential transcription units, the *mamAB* operon, contains the set of essential magnetosome genes sufficient to bring about at least rudimentary magnetosome biomineralization (15). Comparison of (meta)genomes from cultured and uncultured MTB species revealed lineage-specific variations in MAI architecture; however, a small set of core genes (*mamABEIKMOPQ*), largely congruous with the content of the *mamAB* cluster, is conserved across the broad MTB diversity (17–20). For Rhodospirillum rubrum (21) and a hitherto nonmagnetic *Magnetospirillum* sp. (22), it could be demonstrated that it is possible to convey the capability for magbiosyn by transfer of the five biosynthetic gene clusters identified in the MAI of M. gryphiswaldense and related MTB. This, among other hints, lends support to the hypothesis that the magbiosyn trait may have been propagated by horizontal gene transfer (19, 20). However, repeated attempts to achieve magnetization of other foreign organisms by transplantation have failed so far (M. V. Dziuba and D. Schüler, unpublished data). This strongly suggests that there must be additional, auxiliary determinants in the genome that allow proper use of the MAI genes in the first place. In fact, several earlier studies on M. gryphiswaldense have identified functions encoded outside the MAI that are important for magnetosome formation, for instance, genes involved in redox control during aerobic (23) and anaerobic (i.e., denitrifying) respiration (24, 25), as well as in iron reduction (26) and iron homeostasis (27). In addition, a global regulator of carbon metabolism has been assigned a new role in control of magbiosyn in M. gryphiswaldense (28). Yet, how magnetosome formation is integrated into the network of cellular pathways of MTB remains poorly resolved. It is likely that further auxiliary functions are required for this process, possibly regarding membrane biosynthesis capacities and modalities or maturation of proteins and specific cofactors as well as activity modulation of proteins by chaperonins. For a complete understanding of the complex process of magnetosome biosynthesis, it is fundamental to identify all the auxiliary genes that define a genetic background supportive for the expression of the magbiosyn trait. In this context, it is also an important question whether further essential magbiosyn genes outside the MAI can be retrieved.

In MTB, the profound gain in knowledge on biosynthetic determinants and their function in magnetosome formation has been mainly accomplished through reverse genetics and candidate approaches. In contrast, only few studies based on unbiased genome-wide forward methods have been undertaken. One of such well-established and unbiased techniques for identification of a comprehensive set of genes involved in a certain phenotype/pathway is transposon (Tn) mutagenesis. In this forward genetic method, transposons are used to randomly interrupt genes genome wide, and a suitable screening procedure is deployed to select mutant phenotypes indicating impairment of the pathway under study. Generally, the application of such mutagenesis approaches in MTB has been complicated by a number of specific challenges. Thus, most MTB are recalcitrant to grow and be manipulated, genetic tools for high-throughput approaches are limited, there are only inefficient screening methods for the assessment of subtle magnetosome mutant phenotypes, and there is an inherent genetic instability of the magnetic phenotype that leads to spontaneous loss of (parts of) the MAI, with a particularly high frequency under stress conditions (12, 29). A number of transposon mutagenesis studies have been published for the alphaproteobacterium Magnetospirillum magneticum AMB-1 in the past, using Tn*5* (30, 31) or a hyperactive mariner transposon (32, 33). In these studies, a rather limited number of mutants has been screened, ranging from several hundred (32) to a few thousand clones (30, 31, 33), aimed at the identification of mutants entirely devoid of magnetosomes but not considering identification of subtler magnetosome phenotypes. Suspiciously, despite that, some of the studies failed to retrieve essential key genes (30, 31), which were later detected as part of the MAI by reverse genetics. Komeili and coworkers analyzed two unique nonmagnetic mutants for which insertion mapped to magnetosome genes (32). A study by Nash reported that approximately 90% of the nonmagnetic mutants identified were due to spontaneous mutations, and the majority of truly non- or partially magnetic mutants showed an insertion in the MAI. In five cases, genes outside the MAI were affected, two of them encoding redox proteins and one a transcriptional regulator (33). In a more recent nonexhaustive analysis using UV/chemical mutagenesis to identify genes involved in magbiosyn in the emerging MTB model organism Desulfovibrio magneticus RS-1, six mutant alleles located in magnetotactic gene clusters were identified along with four outside these regions, among them two genes encoding ion transporters (34).

In M. gryphiswaldense, another important model for studying magbiosyn, systematic transposon mutagenesis to identify genes involved in magbiosyn has not been conducted so far. This was the aim of the present genome-wide Tn*5* insertion mutagenesis study. We favored conventional Tn mutagenesis over transposon insertion sequencing (Tn-seq) (35), since the magnetic phenotype conferred by the complex magnetosome organelle is only poorly linked to fitness under lab growth conditions. Thus, we needed a more direct proxy than growth fitness for the screening of insertion mutants. The main technical challenges were the achievement of a suitable transposition efficiency and the development of a sensitive screening approach practical for large numbers of clones. After solving these problems, we generated and screened a library of 80,000 transposon insertion mutants. From that, we retrieved 195 stable weakly magnetic or nonmagnetic alleles. The majority of the nonmagnetic mutants had hits within the MAI, whereas most of the weakly magnetic mutants were struck in genes outside the MAI. Among those were several genes already previously linked to magbiosyn, but the majority represented novel potential determinants for magbiosyn. In total, we identified 85 genes outside the MAI for which transposon insertion resulted in a distinguishable but moderate decrease of the ability of mutant cells to biomineralize regular magnetite crystals. These genes may, therefore, encode auxiliary functions for magbiosyn.

## RESULTS AND DISCUSSION

It was expected that inactivation of auxiliary genes would evoke a rather weak impairment of the magnetotactic trait. Therefore, our experimental approaches faced the challenge of discerning subtle mutant phenotypes during the screening process. Two steps had to be optimized. The transposition efficiency had to be maximized to generate a sufficiently large number of transposon clones. Then, a method suitable for the discrimination of subtle differences in the magnetic phenotype (gradually from weakly magnetic to nonmagnetic) and practical for the screening of thousands of clones had to be devised.

### Development of a reliable screening procedure for the mass identification of mutants impaired in magnetosome biosynthesis.

**FIG 1:**
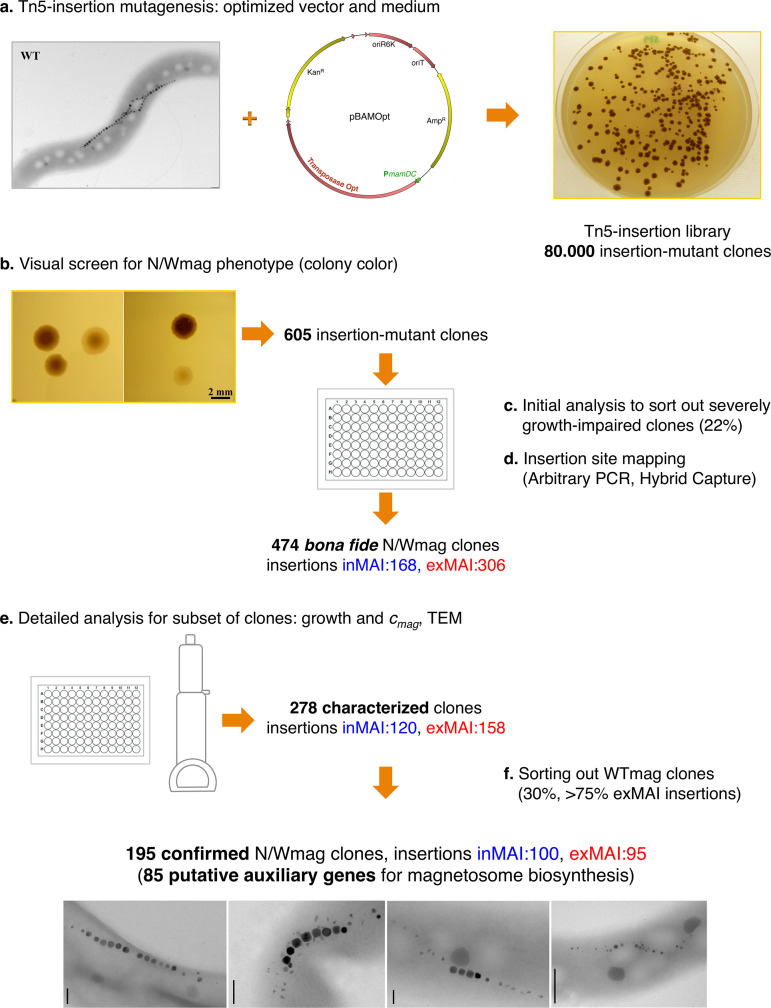
Generation and screening of a genome-wide Tn*5* insertion library of M. gryphiswaldense. The experimental approach, workflow, and yield of insertion mutants are shown. Details on steps a to f are given in the text.

**FIG 2:**
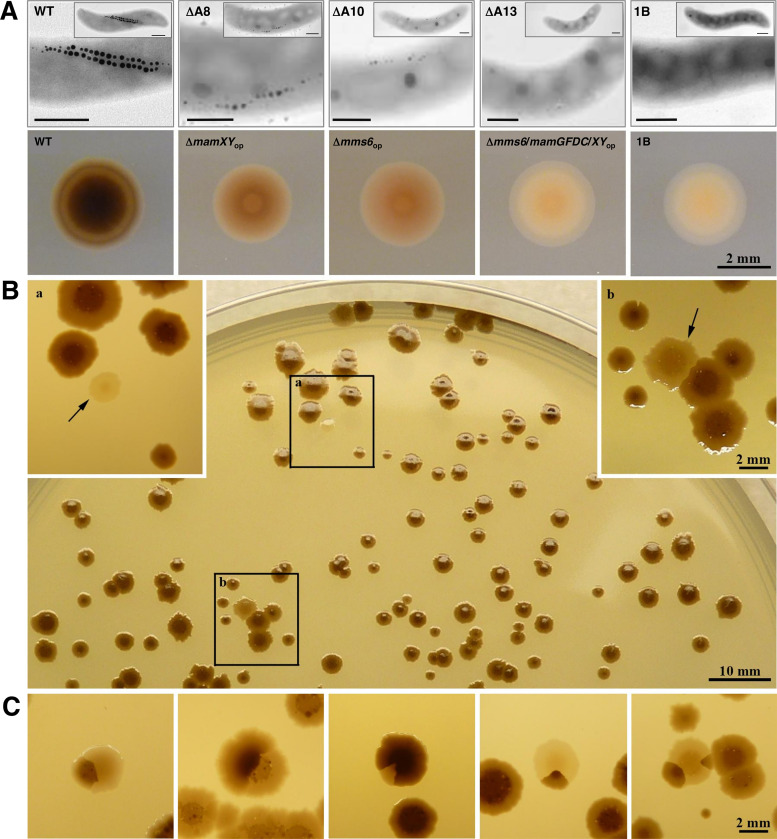
Resolution of the Wmag/Nmag screening procedure demonstrated by examples of different colony appearance. (A) Transmission electron microscopic (TEM) phenotype of magnetosome mutants generated previously and their correlated individual colony appearance. From left: cells of M. gryphiswaldense wild type, cells of M. gryphiswaldense deletion mutants ΔA8, ΔA10, and ΔA13 (15), and cells of a spontaneous MAI deletion mutant (MSR-1B) (13). Scale bars (top), 400 nm. Mid-cell magnifications are shown together with small insets of the respective whole-cell image. Wild-type colonies are dark brown, Δ*mamXY*_op_ and Δ*mms6*_op_ colonies show intermediate color, and the 1B and Δ*mms6*-Δ*GFDC*-Δ*XY*_op_ colonies are cream colored and translucent. op = operon. (B) Agar plate with colonies of M. gryphiswaldense transposon clones grown on ICFM medium for 14 days at 28°C under anoxic conditions. Large dark brown colonies are interspersed with colonies of lighter brown to cream color. The insets show dark magnetic colonies and a whitish colony of an Nmag clone (arrow) (a) or a light brown colony of a Wmag clone (arrow) (b). (C) Aberrant colony phenotypes that were occasionally observed, including twinned, split, or sectored colonies composed of magnetic and non/weakly magnetic cells. This interesting phenomenon might be caused by a fluctuation of phenotypes (transient loss or gain of magnetic phenotype due to regulatory effects).

First, we sought to effectively identify—against the background of cells with wild-type magnetic (WTmag) properties—rare mutants suffering to different degrees from defects in magnetite biomineralization, i.e., cells with diminished magnetic (Wmag) phenotypes, producing fewer, smaller, or aberrantly shaped magnetosomes as well as cells with an entirely nonmagnetic (Nmag) phenotype . Microscopic characterization and the determination of *c_mag_* (i.e., a proxy for the average magnetic orientation of bacterial cells in liquid medium based on light-scattering [36]) are not practical for screening large numbers of samples. We also found that methods employed in earlier forward genetics studies on MTB, such as magnetic depletion by passage through magnetized columns (34) or visible accumulation of cell pellets in 96-well plates exposed to magnets (32, 33), did not provide the sensitivity to discern subtle differences in magnetic phenotypes. Similarly impractical was the use of a range of other phenotypic proxies (data not shown), such as reduced cellular iron content and magnetic distortion of colony shape (37). In contrast, a known characteristic of M. gryphiswaldense that can be easily assessed by visual inspection is the color of magnetic versus nonmagnetic colonies on solid media, with magnetic cells having a darker brown colony appearance due to the black color of intracellularly accumulated magnetite and nonmagnetic cells forming whitish colonies (12, 13, 29, 38, 39). Typically, colonies on these solid media are small, cells form only few magnetosomes, and the use of nontranslucent media such as activated charcoal agar (38, 39) makes it difficult to resolve subtle differences in colony color. However, by testing a range of medium compositions and incubation regimes, we found a substantial increase in colony size (up to 4 to 5 mm, typically 2.5 mm) on large plates (15 cm) with increased medium volume (140 ml versus 100 ml, yielding a thicker agar layer) and increased iron concentration (500 μM versus 50 μM). Prolonged micro- or anoxic incubation (>14 days, optimally 20 days) at lower temperature (<28°C) was found to maximize the expression of the magnetic phenotype, intensifying the colony color due to increased magnetite biomineralization to a dark brown that could be easily recognized on translucent medium (Fig. 1 and 2). Generally, colony size and color were significantly enhanced by low seeding density (ca. 100 colonies per 15-cm plate, i.e., ca. 1 to 2 colonies/cm^2^).

To validate our screening method, we plated single colonies of mutant strains, Δ*mamAB*, Δ*mamXY*, Δ*mms6*, and Δ(*mms6*-*mamGFDC*-*XY*) operon mutants, with well-described impairments in magnetosome biomineralization (15). They cover phenotypes ranging from WTmag to Wmag with gradually reduced magnetosome sizes and numbers and Nmag. Even weak mutant phenotypes could be easily distinguished by different colony colors (Fig. 2) and were recovered with >90% efficiency when mixed with wild-type cultures in spiking experiments (see Fig. S2 in the supplemental material).

10.1128/mSystems.00565-20.6 FIG S2 Results of spiking experiments, performed to screen for defined Wmag mutants with various magnetosome phenotypes in a background of WT cells. Cells of different M. gryphiswaldense deletion mutants were mixed with WT cells (1:99 ratio). Mixtures were plated and colonies tentatively scored by colony color after incubation for 14 days. Ninety-six colonies from each experiment were picked for their putative Wmag phenotype. Their genotypes were determined by PCR bridging across the known deletion site to identify false-positive clones (i.e., erroneously picked as mutant). For comparison, 96 randomly picked WT-like colonies were analyzed in the same way to identify putative false-negative clones (i.e., not recognized as mutant). The diagram shows the percentage of false-negative and false-positive clones recovered in these experiments. Download FIG S2, PDF file, 0.05 MB. Copyright © 2020 Silva et al. 2020 Silva et al. This content is distributed under the terms of the Creative Commons Attribution 4.0 International license.

### Construction of a highly active transposon delivery vector.

In earlier MTB transposon mutagenesis experiments, frequencies of transposition with Tn*5* varied from 1.9 × 10^−4^ (40) to 2.7 × 10^−7^ (30) in AMB-1, whereas for M. gryphiswaldense, insertion frequencies of 10^−4^ to 10^−5^ per recipient have been reported (pSUP1021) (38). However, we found insertion frequencies from different Tn*5* vectors tested under high-throughput conditions to be very low (<10^−8^), fluctuating, and poorly reproducible. We therefore engineered a broad-range Tn*5*-based transposon vector (pBAM1) (41, 42). To enhance expression of the Tn*5* transposase, the respective pBAM1 gene (55% G+C content) was replaced by a synthetic allele that was codon optimized for expression in M. gryphiswaldense (62.8% G+C content) and placed under the control of the strong native *mamDC45* promoter (42) in the vector pBAMOpt (Fig. 1a). In pilot matings, this optimized plasmid yielded a reproducibly increased transposition frequency of approximately 2 × 10^−5^, and Tn*5* insertions in 70 randomly selected clones were found to be distributed fairly randomly across the entire genome (data not shown).

### Generation and screening of a genome-wide Tn*5* insertion library.

For construction of a genome-wide M. gryphiswaldense Tn*5* insertion library (for the experimental work flow, see Fig. 1), we performed seven independent mating experiments for conjugational transfer of the optimized pBAMOpt vector in order to maximize the number of independent Tn*5* insertants. To also allow growth of mutants potentially affected in aerobic or anaerobic respiration, mating reactions were split and incubated under either anoxic or microoxic conditions for 2 to 3 weeks. Overall, this resulted in a Tn*5* insertant library of approximately 80,000 kanamycin (Km)-resistant M. gryphiswaldense clones (Fig. 1a). The phenotypic screening procedure for Wmag and Nmag clones (Fig. 1b to f) consisted of several steps. The initial screen by visual inspection yielded 605 colonies of conspicuous color, representing putative magbiosyn mutants (Fig. 1b). Among them, initial growth analysis in 96-well plates revealed approximately 22% severely growth-impaired clones. These were sorted out, assuming that their apparent magnetic deficiency could be a secondary effect of reduced viability (Fig. 1c). The residual 474 clones were considered bona fide magnetosome mutants (Fig. 1d). Anecdotally, we observed that some clones with a clear N/Wmag phenotype on plates reverted to WTmag upon passaging in liquid culture. This prompted us to conduct a more detailed analysis for a representative fraction (278 clones) of the 474 bona fide mutants (Fig. 1e), for which the initial screen was followed by two passages in liquid culture under microoxic conditions, in order to systematically reassess growth and magnetic response (*c_mag_*) of the clones and to identify potential false-positive clones (i.e., those for which the N/Wmag phenotype was not stable). During all subcultivations, we carefully sought to avoid prolonged stationary growth and storage as well as oxidative stress, since these conditions were previously suspected to induce spontaneous loss of the magnetosome phenotype caused by endogenous transpositions as well as chromosomal deletions and rearrangements within the MAI (12, 13, 29). We found wild-type-like growth in 65% of the 278 analyzed N/Wmag mutants, while approximately one-third (35%) of the clones exhibited moderate growth deficiencies. Clones that displayed a *c_mag_* lower than 80% of the wild type after two passages in liquid medium were considered Wmag, and clones with a *c_mag_* of 0 were considered Nmag. A wild-type-like *c_mag_* (≥80% of wild type [+++]) was shown by 30% (Fig. 1f), a nonmagnetic phenotype (−) by 31%, and a weak magnetic response (between 40% and 80% of wild type [++] or <40% of wild type [+]) by 39% of the clones. The corresponding mutant cells displayed a variety of phenotypes, with magnetosomes being entirely absent (Nmag), reduced in size and/or number, and/or of misshapen appearance (Table 1).

**TABLE 1:**
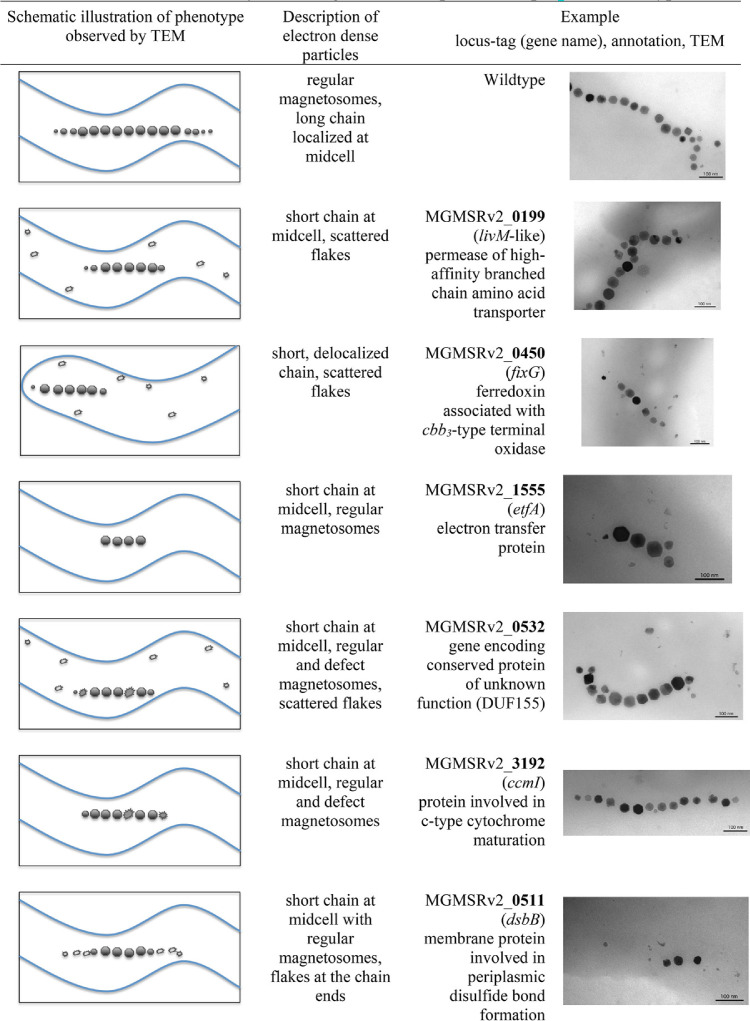
Ultrastructural analysis of magnetosomes in magnetosome mutants: particle shapes^*a*^ and chain-types

a Dark-colored hexagons: regularly shaped and sized, WT-like magnetosomes; dark-colored spiny shapes: irregular magnetosomes; smaller light-colored shapes: thin irregularly shaped, sometimes needle-like particles (flakes) or other aberrantly shaped structures. Selected Wmag clones with Tn*5* insertions outside MAI (exMAI) in genes of different functional categories (electron transport, cytochrome *c* maturation, structural disulfide bond formation, amino acid transport) were analyzed by TEM analysis. Here, we summarize the range of typical aberrations with respect to magnetic crystal size and/or number that we found in cells with a Wmag phenotype compared to the that for the wild type. In most cases, cells show shorter magnetosome chains with wild-type crystal morphologies interspersed by more or less defect crystal shapes and/or scattered flakes (80). Also, nearly regular chains with two distinct crystal types were observed. Magnetosome chains in the mutant cells typically retained their centered location but occasionally were also found delocalized at one of the poles. Apart from deviations in magnetosome structure and positioning, we observed general morphological peculiarities, such as aberrant cell shapes and sizes, or larger polyphosphate granules among Tn*5* insertion mutants, but these are not described or quantified here.

### (i) Clones with unstable, ambiguous, or false-positive phenotypes.

While, for more than two-thirds of the analyzed 278 clones, the bona fide Nmag/Wmag phenotype was confirmed, for almost one-third of them, the magnetosome phenotype proved to be unstable or absent. Although we did not evaluate all 474 bona fide N/Wmag clones by these rather laborious tests, we assume that the observed trend is likely to hold also for the residual set of 196 mutants.

In the 83 “revertant” clones, insertions mapped to genes within the MAI (inMAI) in 24% of the cases (20/83) and to genes outside the MAI (exMAI) in 76% of the cases (63/83). The observed phenomenon of phenotype reversion might be due to one or more of the following reasons. First, a small proportion of Km-resistant colonies might have descended from more than a single cell, giving rise to mixed phenotypes in one apparent colony (Fig. 2C). Second, (some of) these clones may represent “false positives” of our screen for N/Wmag mutants. The rather weak magbiosyn impairment expected from mutations in exMAI genes likely resulted in more subtle deviations from wild-type colony appearance, increasing the probability of misjudging the true magnetosome phenotype. This explanation would be in accordance with the observed doubled frequency of “reversals” in the set of clones where Tn*5* insertion maps to genes outside the MAI (63/158 exMAI clones [40%]) compared to 20/120 (17%) inMAI clones. For example, during several independent rounds of transposon mutagenesis and screening, we retrieved a sometimes conspicuously high number of hits to genes encoding potential functions in cell wall biosynthesis/modification (see Fig. S3). However, null mutants of the respective genes/operons (deletion ranges shown in Fig. S3) displayed a wild-type-like rather than a N/Wmag phenotype in *c_mag_* and transmission electron microscope (TEM) analysis (T. Zwiener, F. Mickoleit, M. Dziuba, C. Rückert, T. Busche J. Kalinowski, D. Faivre, R. Uebe, and D. Schüler, under review). Thus, they likely represent false positives that were erroneously selected during the initial screen as N/Wmag mutants due to a potential change in colony appearance caused by an altered cell surface.

10.1128/mSystems.00565-20.7 FIG S3 Molecular organization of potential “false-positive “gene clusters which received more than two Tn*5* insertions. Mutants with Tn*5* insertion in these genes were picked in the initial visual screen because of aberrant colony appearance but showed no impaired magnetic phenotype in later analyses. For instance, no negative effect on magbiosyn was observed in the deletion mutant of a gene with multiple Tn*5* alleles, which is potentially involved in modification of membrane lipids (MGMSRv2_4002). Likewise, deletion of a huge open reading frame (31 kbp) encoding a potential surface protein with repetitive structure (MGMSRv2_0149) caused only a slight reduction in *c_mag_*, as did the deletion of a cluster of four genes (MGMSRv2_3373 to -3376) potentially involved in cell wall biosynthesis. Tn*5* insertion sites are indicated by arrows (green, WTmag; grey, not magnetically characterized; pink, clones at same hit position present as WTmag or Wmag) and arrowheads (pink, Wmag). Deletion ranges in deletion mutants are indicated by blue bars above the respective cluster. Availability of TEM images for transposon clones is indicated as well as the respective magnetic phenotype of the constructed null mutants. Download FIG S3, PDF file, 0.2 MB. Copyright © 2020 Silva et al. 2020 Silva et al. This content is distributed under the terms of the Creative Commons Attribution 4.0 International license.

Third, the observed “reversal” to wild-type magnetic properties could also be indicative of an underlying regulatory phenomenon resulting in heterogeneity within supposedly clonal cells of a colony or population. Thus, it is possible that a subset of cells reversibly reduces or shuts down magnetosome biosynthesis either stochastically or in response to unknown stimuli, in which case, they would display a N/Wmag phenotype in the screen. The observation of color-sectored “split” colonies consisting of magnetic and nonmagnetic cells (Fig. 2C) seems to be consistent with this assumption. A similar observation of “false-positive” Nmag mutants in *D. magneticus* RS-1 was interpreted as being due to either a proportion of Nmag cells naturally occurring in RS-1 cultures or to lagging expression of the magnetic phenotype in cells after iron starvation (34). This phenomenon of unstable magnetic characteristics may, therefore, be a more common but not yet appreciated feature in MTB.

Another conspicuous observation of the present study is the recovery of a small fraction of mutants (10 of 95 confirmed magnetic mutants in exMAI genes [11%]) (Fig. 1) that were permanently devoid of magbiosyn due to Tn*5* insertion in genes outside the MAI. Their nonmagnetic behavior seemed to be caused neither by severe metabolic impairment, as only minor growth defects were observed, nor by second site mutations in the MAI. The latter was verified for two randomly selected mutants by whole-genome analysis, which confirmed single Tn*5* insertions in two different genes (putative transport protein MGMSRv2_2042 and a beta-ketoacyl synthase domain protein MGMSRv2_1257) (see Table S2) apart from minor sequence alterations (single nucleotide polymorphisms [SNPs]) in several accessory genes of the MAI and some genes outside the MAI. The nonmagnetic phenotype of the 10 exMAI clones seemed to suggest an auxiliary, possibly even essential, role of the affected genes in magbiosyn. However, this appears unlikely, since the Nmag phenotype could not be confirmed in the corresponding unmarked deletion mutants that we constructed (in one case, for MGMSRv2_3634 encoding malic enzyme, the construction of a null mutant turned out to be impossible). Instead, five of the null mutants showed wild-type magnetic properties, and four exhibited an only slightly decreased *c_mag_* value. Also, all but one of the deletion mutants (MGMSRv2_2042 encoding the putative transporter) were severely growth impaired. Thus, most probably, the observed nonmagnetic behavior of the Tn*5* insertion mutants was due to polar effects on the expression of downstream genes. Nevertheless, with respect to regulation-dependent instable magnetic phenotypes, it is interesting to note that one of the 10 Nmag/exMAI genes encodes a diguanylate cyclase (MGMSRv2_3633), and another three of these exMAI genes (MGMSRv2_1015, MGMSRv2_2042, and MGMSRv2_3634) are located in putative transcription units with genes encoding diguanylate cyclases. Given the importance of the second messenger c-di-GMP in bacterial signal transduction networks (43), the observed Nmag phenotype may also hint at hierarchical regulation processes acting on magbiosyn on/offset upon unknown stimuli (potentially only present under screening conditions). Future research is needed to show the role of these candidate auxiliary genes in magbiosyn.

10.1128/mSystems.00565-20.2 TABLE S2 Tn*5* insertion clones with N/Wmag and WTmag phenotype (A) and remaining bona fide N/Wmag Tn*5* insertion clones (B). Download Table S2, PDF file, 0.2 MB. Copyright © 2020 Silva et al. 2020 Silva et al. This content is distributed under the terms of the Creative Commons Attribution 4.0 International license.

### (ii) Tn*5* insertions in genes of the magnetosome island.

**FIG 3:**
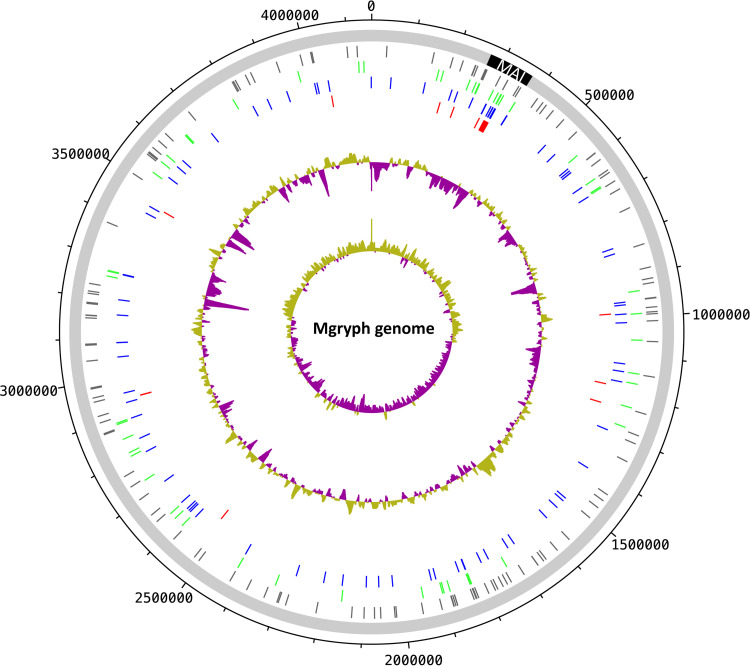
Distribution of Nmag/Wmag Tn*5* insertion sites across the genome of M. gryphiswaldense (Mgryph). Ticks (in circles from outside to inside): gray, insertion sites of clones that have been characterized by colony color only; green, insertion sites in WTmag clones; blue, insertion sites in Wmag clones; red, insertion sites in Nmag clones; %(G+C), with values greater than the average GC content in purple and lower than the average in green; GC skew, with values greater than 0 in purple and values less than 0 in green.

Figure 3 shows the distribution of mapped Tn*5* insertion positions across the M. gryphiswaldense genome.

In 168 (35%) of the 474 bona fide N/Wmag mutants, Tn*5* insertion mapped to genes within the MAI (inMAI). Notably, the average number of identified Tn*5* hits per gene locus within the 100 kbp of the MAI was nearly three times higher than the average number for the genome (see Fig. S4). Of the 168 inMAI mutants, 120 (71%) were characterized phenotypically in addition to colony color, revealing a stable N/Wmag phenotype for 100 of them (83% of 120 characterized; 77 Nmag, 23 Wmag). This represents approximately 50% of all 195 stable N/Wmag clones and essentially all unambiguous Nmag clones (77/87 [89%]; see above for exceptional cases of putative Nmag clones outside the MAI). In contrast, only 21% (23/108) of all retrieved stable Wmag mutants mapped to the MAI. Since the key determinants for magbiosyn identified to date all reside in the MAI, this result was expected. Nearly all (99%) of the stable nonmagnetic inMAI mutants were hit at different positions within the *mamAB* operon (Fig. S4). Notably, we also retrieved N/Wmag hits in the *mamJ* and *mamK* genes, which do not play a key role in magbiosyn itself but rather in magnetosome chain formation, although it has been shown that deletion of *mamK* results in pleiotropic effects, among them, a significant reduction of magnetosome numbers per cell (44). Also, Tn*5* insertion in *mamJ* or *mamK* may have a polar effect on downstream gene expression of essential magbiosyn genes in the *mamAB* operon.

10.1128/mSystems.00565-20.8 FIG S4 Distribution of Tn*5* insertions within genes of the MAI. Vertical arrows represent Tn*5* insertions identified in the genes of the MAI, with arrow length symbolizing the number of Tn*5* hits according to the unit arrow length given in the legend (red, per gene; blue, per kilobase of gene). The height of the columns given in grey represents the fraction of Tn*5* insertants that has been characterized regarding growth and *c_mag_*, with black bars standing for the fraction of Nmag, hatched bars for the fraction of Wmag, and open bars for the fraction of WTmag phenotype of the characterized clones. Gene functions are coded by colored underlining according to the main phases of magnetosome biosynthesis and chain formation: blue (1), invagination of magnetosome membrane (MM) to form vesicles; green (2), recruiting of further proteins to the MM; orange (3a), iron transport into the vesicles; yellow (3b), precipitation of iron in the vesicles; pink (3c), control of Fe^2+^/Fe^3+^ ratio within the vesicles; blue (3d), magnetite nucleation; black (3e), crystal maturation as well as crystal size and shape control; dark green (4), chain formation and positioning. Genes in the *mamAB* operon that are conserved across MTB genomes are underlined by a thick red line. Schematic representation of MAI and protein domain coloring according to reference 10. Download FIG S4, PDF file, 1.7 MB. Copyright © 2020 Silva et al. 2020 Silva et al. This content is distributed under the terms of the Creative Commons Attribution 4.0 International license.

Some of the MAI genes represented Nmag as well as Wmag mutant alleles (*mamB*, *mamI*, *mamO*, *mamK*, and *mamN* [number of Nmag ≫ number of Wmag]; *mamA* [number of Nmag > number of Wmag]; *mamP* [number of Nmag < number of Wmag]). Several MAI genes showed only Wmag mutant phenotypes (*mms6*, *mms36*, *mmsF*, *mamH*, *mamR*, *mamZ*, *mamX*, *feoA1*, and *feoB1*). Neither Wmag nor Nmag mutants were identified in *mamU*, *mamY*, *ftsZ*-like, or, notably, any of the genes of the *mamGFDC* operon. This is consistent with known weak phenotypes of targeted gene deletions (15, 16, 45, 46).

Tn*5* insertion in essential genes of the *mamAB*_op_ of the MAI led to a Wmag instead of a nonmagnetic phenotype in 14% of clones or had no effect on magnetic properties at all (WTmag) in 12% of the clones. This observation may be surprising at first glance. However, it is possible that Tn*5* insertion in these cases affects only certain domains of the gene products, leading to the expression of truncated but at least partially functional proteins. Alternatively, transcriptional readthrough may occur, giving rise to the same effect. Also, in putative transcriptional units, expression of genes downstream of those affected by Tn*5* insertion may still be possible if transcription commences from internal, so far unknown promoters.

### (iii) Tn*5* insertions in genes outside the MAI.

For 306 (65%) of the 474 bona fide N/Wmag clones, Tn*5* insertions mapped to genes outside the magnetosome island (exMAI). Of these, 158 (52%) were characterized in more detail, yielding 95 Tn*5* insertants (60%) with a confirmed N/Wmag phenotype. These 95 hits correspond to a set of 85 exMAI genes which comprise a pool of putative auxiliary functions for magnetosome biosynthesis (Fig. 1f) (the small fraction of 11% nonmagnetic mutants affected in genes outside the MAI was described above). The majority of the stable magnetosome mutants with Tn*5* insertion in exMAI genes displayed a Wmag phenotype (85/95 [89%]). exMAI Wmag mutants also represent the dominant fraction of the total of recovered stable Wmag mutants (85/108 [79%]). Since a high frequency of spontaneous deletions in the MAI has been reported for M. gryphiswaldense (12, 13), we verified the integrity of the essential *mamAB* operon by PCR amplification for all phenotypically characterized mutants where Tn*5* insertion in an exMAI gene was mapped by arbitrary PCR (ARB-PCR) (selected subset of 72/158 exMAI mutants) (data not shown).

### (iv) Wmag mutants affected in genes outside the MAI.

Several functional categories of genes were frequently found in the overall pool of exMAI Tn*5* insertants (verified N/Wmag and WTmag, respectively) (Table S2A) (residual bona fide N/Wmag) (Table S2B). These comprise genes involved in (i) redox reactions (e.g., electron transport, cytochrome *c* maturation, and nitrite and nitric oxide reduction), (ii) sulfur metabolism (e.g., cysteine biosynthesis and disulfide bond formation), (iii) signal reception/transduction and chemotaxis/motility, (iv) membrane transport, (v) nitrogen metabolism, (vi) regulation of gene expression, and (vii) fatty acid/lipid metabolism. Strikingly, a number of pathways received multiple hits of Tn*5* insertion, which are explained in detail below. In addition, we recovered some genes of central and carbon metabolism as well as numerous genes encoding conserved proteins of unknown function, among them, some transmembrane proteins, exported proteins, and tetratricopeptide repeat (TPR) containing proteins potentially mediating protein-protein interactions.

To assess whether certain classes of gene functions are characteristic for the set of exMAI Tn*5* hits identified here, we compared the gene product annotations of exMAI Tn*5* hits to those of the whole M. gryphiswaldense proteome in a gene ontology (GO) term enrichment analysis (Fisher’s exact test [FET]) (see Fig. S1). The test set contained all 75 exMAI Tn*5* insertants with a stable Wmag phenotype, whereas the reference set consisted of the remaining protein-coding sequences of the genome (3,717 genes, 123 MAI genes not included). The FET analysis revealed 57 GO terms significantly overrepresented (*P* value < 0.05) in the set of Tn5 hits, which correspond to 36 genes (see Table S3). It is noteworthy that among the overrepresented GO terms, “oxidation-reduction process” and “protein histidine kinase activity” are linked to a conspicuously high number of genes within the test set of magnetosome impaired Tn*5* hits (13 and 6 of 36 genes, respectively), indicating that redox reactions and signal transduction events may be of particular importance in support of magbiosyn.

10.1128/mSystems.00565-20.3 TABLE S3 ExMAI N/Wmag genes associated with overrepresented GO terms identified by FET analysis against the genomic background. Download Table S3, PDF file, 0.03 MB. Copyright © 2020 Silva et al. 2020 Silva et al. This content is distributed under the terms of the Creative Commons Attribution 4.0 International license.

10.1128/mSystems.00565-20.5 FIG S1 Schematic outline for GO term enrichment tests with different pairs of gene sets from M. gryphiswaldense. Gene sets include the genome (less MAI, large oval), the set of exMAI Tn5-hits that lead to Nmag/Wmag phenotypes (small oval), the MAI (large circle), and the set of inMAI Tn5-hits that lead to Nmag/Wmag phenotypes (small circle). GO-term sets extracted from the different gene sets are depicted by squares and triangles. Extract from the genome: large square, from the MAI: small square, from the exMAI Tn5-hits: large triangle, from the inMAI Tn5-hits: small triangle. Download FIG S1, PDF file, 0.4 MB. Copyright © 2020 Silva et al. 2020 Silva et al. This content is distributed under the terms of the Creative Commons Attribution 4.0 International license.

**FIG 4:**
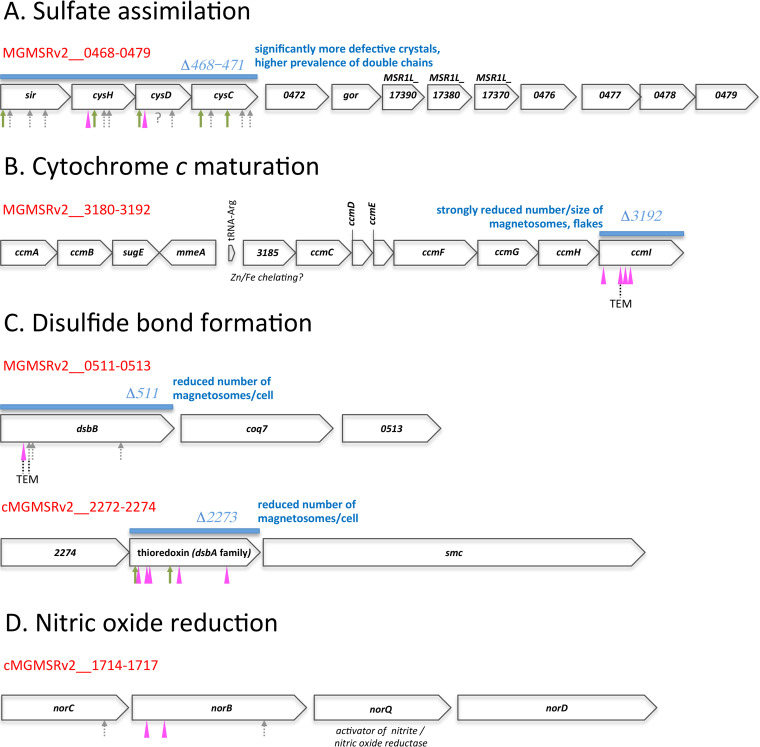
Molecular organization of gene clusters which received more than two Tn*5* insertions and are correlated with magnetosome mutant phenotypes. (A to D) Gene clusters are delineated by locus tag ranges (in red above each cluster, corresponding to the MSR-1 v2 genome, MGMSRv2__[number]; “c” means encoded on complementary strand). Open reading frames are labeled either with gene name or with corresponding locus tags of the MSR-1 v2 genome (number only) or the R3/S1 genome (MSR1L_[number]). Tn*5* insertion sites are indicated by arrows (green, WTmag; gray, not magnetically characterized; pink, clones at same hit position present as WTmag or Wmag; ?, unknown insertion position) and arrowheads (pink, Wmag). Deletion ranges in deletion mutants are indicated by blue bars above the respective cluster. Availability of TEM images for transposon clones is indicated as well as the respective magnetic phenotype of the constructed null mutants.

Recently, a number of studies suggested that proteins encoded outside the MAI are potential auxiliary players in magnetosome formation, among them, specific redox-active enzymes. Some of them, such as nitric oxide reductase Nor (24), cytochrome *cd*_1_ nitrite reductase Nir (25), and oxidases such as terminal oxidase Cbb3 (an oxygen sensor [23]), were also part of the potential auxiliary gene set delineated in our present transposon mutagenesis study (Nor, Fig. 4D; Table S2A and B) (Nir and Cbb3, Table S2A and B). This corroborates their potential supportive function in magnetosome formation. In contrast, other genes encoding redox enzymes implicated in the process earlier, for instance, two types of ferric reductase (26), the periplasmic nitrate reductase Nap (24), or regulators such as the ferric uptake regulator (*Mg*Fur, MGMSRv2_3137) (27) and the oxygen sensor *Mg*Fnr, (MGMSRv2_2946, gene *aadR*) (47), have not been retrieved as magnetically deficient Tn*5* insertion clones in our analyses, possibly due to our less-than-fully saturated screen (86% as determined according to the Poisson distribution from the number of Tn*5* hits per gene locus). Interestingly, a different type of regulator, the global carbon metabolism regulator Crp, has recently also been implicated in magbiosyn. Deletion of MGR_1896 [MSR1(L)_26600], encoding a member of the Crp family, has been found to impair magbiosyn, leading to strongly decreased iron content of the cells and misshapen magnetosome crystals (28). Here, we identified a gene encoding a further member of the Crp/Fnr family as a bona fide Wmag Tn*5* insertion (MGMSRv2_1404), which has also been found among the differentially expressed genes in a recent transcriptomics study (upregulated under low-oxygen conditions [48]). These findings suggest a link between global carbon metabolism and the energy-consuming process of magbiosyn in the MTB cell. Further notable instances, where the present results are in accordance with earlier suggestions of an auxiliary role in magbiosyn, belong to the class of membrane transporters. Thus, we recovered a N/Wmag Tn*5* insertion in a gene encoding a TauE-like transport protein (MGMSRv2_1267) that belongs to the same family as a protein from *D. magneticus* RS-1, whose encoding gene has been found to yield Nmag cells upon mutation (34). Another potential transport protein, recently identified as a novel candidate for a true magnetosome membrane protein in a proteome analysis by (49), was also part of the N/Wmag gene set identified in our screen (MGMSRv2_3281). This protein belongs to a Bax1 inhibitor family (PF01027; http://pfam.xfam.org/), and a bacterial member of this family has been shown to function as pH-sensitive calcium leak across membranes (50).

### (v) Gene clusters/pathways with multiple Tn*5* hits.

In the course of seven independent rounds of transposon mutagenesis and screening, we observed a conspicuous accumulation of Tn*5* insertion events in exMAI genes of specific cellular pathways. In Fig. 4A to D, these genes are depicted in their genomic context (potential operons). Notably, N/Wmag and (revertant) WTmag alleles were often found closely adjacent in the same gene. Particularly frequent Tn*5* insertions were found in genes involved in sulfate assimilation, oxidative protein folding, nitric oxide reduction (denitrification pathway), and cytochrome *c* maturation. In the following, we will focus on these pathways.

### (vi) Sulfate assimilation.

We retrieved 17 mutants where Tn*5* insertion mapped in close proximity to one of four adjacent genes of a putative transcription unit involved in sulfate assimilation (Fig. 4A). The accumulation of Tn*5* hits in these four genes is conspicuous, even though it turned out that only two of seven clones that we characterized showed a stable Wmag phenotype. These clones were affected in *cysD* (MGMSRv2_0470) and *cysH* (MGMSRv2_0469) encoding ATP sulfurylase and phosphoadenosine phosphosulfate (PAPS) reductase, respectively. We constructed a markerless deletion mutant of the four-gene operon MGMSRv2_0496 to -0470 (Fig. 4A), which exhibited impaired growth especially under oxic conditions but synthesized magnetosome crystals in approximately normal size and number per cell. However, a decisive difference to wild-type cells was the higher prevalence of cells with double magnetosome chains of reduced length and an almost three times increased fraction of imperfect particles, mostly twinned crystals, in null mutant cells. The generation of reduced sulfur during sulfate assimilation affects cysteine biosynthesis and the formation of crucial electron transfer moieties such as iron-sulfur clusters and, hence, has an impact on electron flow and redox state maintenance. Since these processes are known to play an important role in the formation of magnetosomes (23–25, 51–53), their impairment can be expected to have a negative effect on magbiosyn. Interestingly, the expression of sulfate assimilation genes harbored in the above-described Tn*5* insertion hot spot has been found to be upregulated in a differential expression study upon shift to high-iron conditions in M. gryphiswaldense (54).

### (vii) Cytochrome *c*-type biogenesis pathway.

We recovered four different Tn*5* insertions in a cluster of cytochrome *c* (cyt *c*) maturation genes (Fig. 4B). Notably, they exclusively map to the *ccmI* (*cycH*) gene (MGMSRv2_3192). The cyt *c* biogenesis protein CcmI (CycH) is a tetratricopeptide repeat (TPR)-containing protein thought to act as an apo-cyt *c* chaperone. It is part of the CcmFHI module involved in stereospecific ligation of heme *b* to thiol-reduced apo-cyt *c*. In the cyt *c* maturation system of the alphaproteobacterium Rhodobacter capsulatus, CcmI consists of two segments, the N-terminal membrane-spanning CcmI-1 and the C-terminal periplasmic CcmI-2, which are supposed to have different functions (55, 56): CcmI-2 mediates the electron transfer from the cytoplasm to the thiol-oxidized periplasmic apo-cyt *c*, whereas CcmI-1 is responsible for stereospecific ligation of heme *b* to the thiol-reduced apo-cyt *c*. M. gryphiswaldense CcmI exhibits a bipartite architecture similar to the homologous R. capsulatus protein. All four Tn*5* insertions observed in our study mapped to the N-terminal CcmI-1-segment of the M. gryphiswaldense protein (see Fig. S5) and yielded a Wmag phenotype in the respective insertion mutants. Two of the four insertion mutants of *ccmI*, strains 11 (20/9) and 26 (5/9), were analyzed in more detail. They synthesized a reduced number of particles with smaller diameter (on average seven particles per cell with a diameter of 21 nm), leading to decreased magnetic response compared to that of wild-type cells (*c_mag_* values of 0.96). In TEM images of mutant cells, short magnetosome chains were found, with mature magnetosomes frequently interspersed by misshapen crystals (Table 1). We constructed an unmarked *ccmI* deletion mutant, which was found to be even more deficient in magnetosome formation, although magbiosyn was not completely abolished (see Table S4). It is likely that the observed ultrastructural deviations in the mutant strains are due to an impairment of cyt *c* maturation caused by reduced CcmI activity, since the MAI harbors four *c*-type cytochromes which, by their putative redox capabilities, may have an important role in the process of magnetosome formation (so-called magnetochromes MamP/E/T/X [57, 58]). Given the presence of additional 32 genes encoding *c*-type cytochromes in the M. gryphiswaldense genome, a lower capacity for cyt *c* maturation will likely also inhibit other enzyme systems, for instance, the activity of anaerobic/aerobic respiration enzymes.

10.1128/mSystems.00565-20.4 TABLE S4 Characteristics of M. gryphiswaldense deletion mutants in genes for which Tn*5* insertion yielded a magnetosome phenotype. Download Table S4, PDF file, 0.7 MB. Copyright © 2020 Silva et al. 2020 Silva et al. This content is distributed under the terms of the Creative Commons Attribution 4.0 International license.

10.1128/mSystems.00565-20.9 FIG S5 Selected Tn*5* insertion alleles leading to Wmag phenotypes. (A) Insertion in the *ccmI* gene. MSR1(L)_29190 amino acid sequence; MGMSRv2_3192 insertion positions 3.268.033, 3.268.233, 3.268.257, and 3.268.294 (BLASTp: both sequences are 98% identical with 6 amino acid [aa] differences, no gaps). Reduced numbers of magnetosomes with a smaller diameter have been shown for Wmag alleles 1 and 3. (B) Insertion in the *dsbB* gene. MSR1(L)_17040 amino acid sequence; MGMSRv2_0511 insertion positions 556.222, 556.223, 556.260, and 556.447 [BLASTp: aa sequences of MSR1(L)_17040 and MGMSRv2_0511 are identical]. In case of the Wmag allele 1, Tn*5* insertion leads to reduced numbers of magnetosomes with a smaller diameter. (C) Insertion in the *dsbA*-like gene. MSR1(L)_22700 amino acid sequence; MGMSRv2_2273 insertion positions 2.403.749, 2.403.925, 2.403.974, 2.404.129, 2.404.131, 2.404.182, and 2.404.184 (BLASTp: both sequences are 99% identical with 3 aa differences, no gaps). Lightning bolts, Tn*5* insertion positions (affected amino acid in pink). Open lightning bolts indicate insertion positions in clones that have been picked up by the visual screen as Wmag but have not been magnetically characterized. Green, Wmag phenotype; pink, WTmag. Topologies as predicted by TMHMM server v 2.0, TM, transmembrane region; sigP, signal peptide as predicted by SignalP 4.1 server. TPR, TPRs as predicted by TPRpred server (thicker lines, lower E values, i.e., higher significance). (A) Red plus grey regions constitute segment 1, the blue region constitutes segment 2 of the CcmI protein (periplasmic region, TPR domain). Leucine-repeats do not correspond to a leucine-zipper according to prediction by the 2ZIP server. Lightning bolts outlined in red indicate alleles with spectroscopically characterized Tn*5* clones (UV/Vis). (C) Functionally important residues are shown in bold (CxHC motif, additional C residues). Download FIG S5, PDF file, 1.1 MB. Copyright © 2020 Silva et al. 2020 Silva et al. This content is distributed under the terms of the Creative Commons Attribution 4.0 International license.

### (viii) Disulfide bond formation pathway.

We recovered 11 Tn*5* mutants with insertions in two genes encoding proteins of the disulfide bond (DSB) pathway of periplasmic oxidative protein folding (Fig. 4C), one encoding a DsbA-like protein (MGMSRv2_2273, affected in 7 of the 11 cases) and one encoding DsbB (MGMSRv2_0511, affected in 4 of the 11 cases). *c_mag_* characterization of the seven *dsbA* alleles showed a stable Wmag phenotype for six of them and also for the one *dsbB* allele tested. As revealed by TEM analysis, cells of the Wmag *dsbB* insertion displayed mid-cell-positioned magnetosome chains with mature crystals but flakes at the chain ends (Table 1). Upon unmarked deletion of the *dsbB* gene, cells of the null mutant (Δ*dsbB*) clearly showed a smaller number of magnetosomes, whereas the size of magnetosomes was not significantly reduced (Table S4). The deletion mutant of the *dsbA*-like gene exhibited very short magnetosome chains, occasionally flakes at the chain ends, or few disconnected crystals or flakes (Table S4).

Together, our results suggest that proper folding of periplasmic proteins by disulfide bond formation is a prerequisite for efficient magnetosome biosynthesis. Indeed, several magnetosome membrane proteins possess more than one cysteine residue and might be substrates of this oxidative folding pathway. In proteins that are exported from the cytoplasm to the cell envelope (periplasm, outer membrane, and extracellular environment), disulfide bond formation is part of a maturation process which contributes to their structural stabilization and, thus, ensures their functionality (59). In Escherichia coli and other bacteria, several periplasmic disulfide bond-forming proteins (thiol-disulfide oxidoreductases which are members of the thioredoxin superfamily and contain pairs of cysteine residues) are involved in DSB. Periplasmic DsbA introduces disulfide bonds in its protein substrate as it is translocated across the cytoplasmic membrane and becomes reoxidized by the cytoplasmic membrane protein DsbB that in turn passes the electrons to the terminal electron acceptor via a quinone. Periplasmic DsbC and DsbG are protein disulfide isomerases that can correct wrongly positioned disulfide bonds in proteins with more than two cysteine residues. DsbC and DsbG are reduced by the cytoplasmic membrane protein DsbD which, in turn, is provided with electrons from the cytoplasmic thioredoxin system (60). In M. gryphiswaldense, DSB seems to comprise homologs of DsbA (MGMSrv2_2273) and DsbB (MGMSrv2_0511) as well as a putative fusion protein of DsbC and DsbD (MGMSrv2_4064). A homolog for DsbG known to protect single cysteine residues in periplasmic proteins from oxidation (61) has not been detected in M. gryphiswaldense. DsbA and DsbB are encoded in separate transcription units (Fig. 4C), suggesting that there are many different substrates for the DSB pathway in M. gryphiswaldense rather than only few specific ones in which case a *dsbAB* operon would have been expected (60).

Whereas in E. coli several DSB protein substrates have been identified (among them, the outer membrane protein OmpA, periplasmic alkaline phosphatase PhoA, the flagellar protein FlgI, the lipopolysaccharide [LPS] assembly protein LptD, the cell division protein FtsN, several lipoproteins, metal transporters, and amino acid/peptide transporters [59]), there are so far no experimentally verified DSB substrates in M. gryphiswaldense. However, several membrane proteins of the MAI may be substrates of this pathway, as they possess two or more (up to eight) cysteine residues. Considering the current magnetosome vesicle formation model (9, 10), MAI membrane proteins exhibiting domains oriented toward the luminal side of the magnetosome vesicle may have been exposed to the periplasm prior to vesicle formation. Where these protein domains contain cysteine residues, they should have been protected from oxidation by the formation of disulfide bonds. MAI membrane proteins that contain more than two cysteines in predicted luminal domains (62) are MamE/F/G/H/N/P/S/T/X/Z (large luminal domains, MamE/P/S/T/X/Z; even number of cysteine residues, MamF/N). If some of these proteins are indeed DSB substrates, impairment of this pathway will have a negative effect on the structural stability and, hence, the abundance of these proteins, which could account for the observed Wmag phenotype of the respective Tn*5* insertion mutants. Furthermore, since several periplasmic thiol-redox reactions of the cytochrome *c* maturation system (involving, for instance, CcmH, CcmG, and apo-cyt *c*) depend on the functionality of DSB (63), and given the special importance of cyt *c* for proper function of the magnetochromes MamP/E/T/X, impairment of DSB can be expected to have a fundamentally disturbing effect on the process of magnetosome formation.

### Conclusions.

In recent years, it has become more and more apparent that the genetic and structural complexity of magbiosyn is larger than originally assumed. It gradually emerges that, apart from the approximately 30 core genes initially thought to orchestrate the magnetic phenotype, there must be many more. We previously observed that transfer of the magbiosyn capability by transplantation of the MAI is possible for certain organisms such as Rhodospirillum rubrum (21) and the nonmagnetotactic *Magnetospirillum* sp. strain 15-1 (22), but it failed for many others tested, including E. coli (M. V. Dziuba and D. Schüler, unpublished data). This leads to the pivotal question of what the supportive functions required for magbiosyn are in addition to known genes of the MAI. Solving this question would enhance our understanding of microbial biomineralization but also bears great relevance for the fields of synthetic biology and biotechnology (64); for instance, it would considerably facilitate approaches for magnetization of other (micro)organisms.

In the present study, we identified 195 M. gryphiswaldense clones compromised in magbiosyn by using a systematic transposon mutagenesis approach. In approximately 50% of the cases, the affected genes were found to be located within the MAI, among them, essentially all of those where transposon insertion yields a stable nonmagnetic phenotype. This underscores the widely proven essentiality of the MAI for the process of magbiosyn and validates our experimental approach. In the other 50% of the N/Wmag genes, encoded outside the MAI, we recovered several that have recently been linked to magnetosome formation as putative supporting determinants, such as nitrate reduction and denitrification (24), thus corroborating the findings of earlier studies and verifying our identification strategy. In contrast to observations reported by earlier studies (12, 13, 33), in none of the tested exMAI Tn*5* insertion clones were spontaneous MAI deletions the reason for the observed magbiosyn impairment. That we failed, on the other hand, to retrieve some of the known auxiliary candidates such as iron reductases or the Fur regulator may be due to the fact that our screen is, as expected, not exhaustive (86% probability that all relevant loci have been detected). Another reason may be that the screening approach is still too insensitive for very subtle mutant phenotypes. Nevertheless, our systematic study presents the so far most comprehensive set of auxiliary gene candidates for magbiosyn. In particular, it newly defines certain cellular pathways as specifically important for magbiosyn that are conserved in MTB but have not been implicated in this process so far, such as periplasmic disulfide bond formation, cytochrome *c* maturation, and sulfate assimilation.

In theory, recent high-throughput specifications of Tn mutagenesis (e.g., Tn-seq [35, 65]) may, by their unbiased high-throughput design, have the potential to yield a more rigorous assessment. Approaches such as Tn-seq have proven to be extremely powerful in delineating complete numbers of alleles involved in several bacterial pathways, e.g., the production of antibiotics (66), sporulation (67), or methylotrophy (68). However, a great advantage of our conventional approach of genome-wide transposon insertion mutagenesis in the search for genes supporting magbiosyn is that it allows direct targeting of growth/fitness-unrelated functions, which cannot be easily selected against in Tn-seq approaches. Also, with our conventional approach, a correlation between pheno- and genotype at the level of clones is possible.

Apart from the result of a manageable pool of putative auxiliary determinants as the basis for further experimental work, there are two main insights from our study. First, and notably, outside the MAI, we could not detect further MTB-specific gene clusters involved in magbiosyn. Rather, the process of magbiosyn seems to be particularly dependent on the function of a number of general cellular pathways; apparently, it is vulnerable if these pathways are impaired. Their genes (i) ensure the proper folding of proteins that directly take part in the process of magbiosyn, (ii) provide the cell with sufficient amounts of redox mediators by affecting their maturation, such as in the case of cyt *c*, or by enabling their biosynthesis through furnishing important amino acids such as cysteine in the case of iron sulfur clusters, (iii) act in/modify cellular nitrogen metabolism, (iv) balance cellular energy metabolism, (v) take part in cell wall biosynthesis/modification (with reservations, since the genes of this category may pop up as false positives in the visual screen because of changes in colony appearance caused by an altered cell surface), and (vi) are responsible for signaling and regulatory cues in the context of magbiosyn. Except for the magbiosyn-specific signaling modules, all of the pathways mentioned above may result in decreased cellular fitness when disturbed. However, insertion mutants affected in these genes were found to grow rather well yet were more or less severely affected in magbiosyn as judged by the structural defects of magnetosomes and chains. Thus, the pathways we identified seem to affect magbiosyn particularly strongly.

Second, our results suggest that regulation of magnetosome formation may be interlaced with cellular state by cues from cellular (energy) metabolism. Transcriptional regulators of specific MAI genes may serve as auxiliary genes, since ill-balanced expression levels might be sufficient to disturb the process of magbiosyn and cause an aberrant magnetic phenotype. An extreme case may be exemplified by nonmagnetic Tn*5* insertions affected in metabolic genes, such as those encoding malic enzyme and others (see above). One could imagine that certain pathways, when impaired, challenge cellular fitness in a specific way, requiring larger efforts of the cell to cope with the corresponding stress situation, thereby leading to a cutdown of cellular resources for magbiosyn as a beneficial but nonvital process and, thus, resulting in a mutant magnetic phenotype. If so, one could expect that magbiosyn as a costly process is not turned on at all to save all resources for stress management. The consequence would be a nonmagnetic phenotype. Future experimental work is necessary to evaluate the hypotheses inferred from the results of the present study. In this context, it would be interesting to also address the question about a (master) regulator(s) for magbiosyn (other than oxygen) that may act as a “switch” integrating different types of cellular information with magbiosyn to regulate on/offset of this costly process depending on the cellular state.

Finally, we close these considerations with a different interpretation of our data. Although specific auxiliary genes for magbiosyn may exist—and several of the genes retrieved in this study might turn out as such—it is also possible that the ground for magbiosyn is prepared by a more general metabolic network rather than by specific single genes. Our observation of basic conserved cellular pathways as particularly relevant for magnetosome formation and the broad spectrum of functions in the delineated set of candidate auxiliary genes support this notion. It would also be in line with the hypothesis of an earlier study proposing that the potential of an organism to synthesize magnetosomes is dependent on a specific metabolic profile (69).

## MATERIALS AND METHODS

### Bacterial strains, plasmids, and growth conditions.

Escherichia coli strain WM3064 was grown in LB medium with 300 μM diaminopimelic acid (DAP) (70). Routinely, bacterial strains were cultivated on solid media with 1.5% (wt/vol) agar. For strains carrying recombinant plasmids, media were supplemented with 25 μg/ml kanamycin (Km) for E. coli WM3064 and 5 μg/ml Km and 30 μg/ml ampicillin (Amp) for M. gryphiswaldense. Bacterial strains and plasmids used in this study are described in Table S1A in the supplemental material.

10.1128/mSystems.00565-20.1 TABLE S1 Bacterial strains and plasmids (A) and primers (B) used in this study. Download Table S1, PDF file, 0.09 MB. Copyright © 2020 Silva et al. 2020 Silva et al. This content is distributed under the terms of the Creative Commons Attribution 4.0 International license.

### Cultivation of Magnetospirillum gryphiswaldense MSR-1.

**(i) Plate cultivation of M. gryphiswaldense cells for phenotypic screening.** In summary, maximum expression of the magnetic phenotype was achieved using the following optimized conditions, which were then consistently applied throughout all subsequent experiments: 140 ml of improved colony formation medium (ICFM), i.e., flask standard medium (FSM [51]) supplied with an increased amount of iron (500 μM), in large-size (150 mm) Petri dishes at low seeding density of a maximum of 100 Km-resistant Tn-insertant colonies per plate (1 to 2 colonies per cm^2^) with an increased incubation time (>14 days) at 28°C under microoxic (2% O_2_ in the headspace) or fully anoxic (100% N_2_ in the headspace) conditions.

**(ii) Liquid cultivation.** M. gryphiswaldense was grown microaerobically in FSM at 30°C with moderate agitation (120 rpm). To record growth curves, microaerobically grown precultures of all strains were inoculated at an optical density at 565 nm (OD_565_) of 0.025 into 3 ml FSM with 8 mM sodium nitrate (oxic, microoxic, and anoxic growth) or 4 mM ammonium chloride instead of sodium nitrate (oxic growth only) in six-well plates with duplicates per strain. Cultures were then incubated for 48 h at room temperature under oxic, microoxic, and anoxic conditions. For oxic conditions, the plates were placed under ambient oxygen concentration, while for microoxic conditions, plates were incubated in metal jars with 2% O_2_ in 98% N_2_. Anaerobic conditions were achieved by incubation in a 100% N_2_ atmosphere in glass jars. OD_565_ was then measured at regular time intervals with an Infinite M200 Pro plate reader (Tecan, Switzerland), shaking the plates for 40 s before each measurement. To avoid disturbance of anoxic conditions, OD_565_ was only measured at 0 and 48 h for anaerobic cultures. At the end of the experiment, aliquots of cultures were taken to analyze cellular magnetic response (*c_mag_*) with an Ultrospec 2100 pro (Biosciences, Amersham) photometer as described previously (36) and to prepare transmission electron microscope (TEM) samples.

Oxygen band formation and gas production were analyzed in oxygen gradient tubes containing FSM with 0.3% agar inoculated with cell cultures from microoxic Hungate tubes. Oxygen gradient tubes were incubated for 48 h at 27°C under atmospheric conditions.

### DNA protocols.

DNA isolation, digestion, ligation, and transformation were essentially according to standard methods (70). PCR products and vector inserts were sequenced using BigDye Terminator version 3.1 chemistry (Applied Biosystems, Darmstadt, Germany) on an ABI 3700 capillary sequencer.

### Construction of the transposition vector pBAMOpt.

To increase transposition frequencies in M. gryphiswaldense, the transposase-encoding *tnpA* gene residing in the engineered mini-Tn*5* transposon vector pBAM1 (41) was replaced by a synthetic codon-optimized allele under the control of a strong native promoter (P*_mamDC45_*) (42), resulting in the plasmid pBAMOpt (Fig. 1). The synthetic transposase gene with the native promoter P*_mamDC45_* was designed and synthesized by GeneArt (Thermo Fisher Scientific) and provided in a standard vector that was transformed into E. coli DH5α. The strain was grown, and the plasmid was extracted and digested with Swa/PmeI. The DNA band of the correct size was then purified and cloned into a linearized pBAM1 devoid of its transposase gene. pBAMOpt with the optimized transposase and native strong promoter was checked for correct sequence.

### Construction of a Tn*5* insertion library.

The pBAMOpt plasmid was used to create random Tn*5* insertion mutants in M. gryphiswaldense. It was mass conjugated from E. coli WM3064 to M. gryphiswaldense wild-type cells as previously described (38), using 10^9^ M. gryphiswaldense cells and a donor/recipient ratio of 1:1. The resulting cell pool was then plated on large selection plates (Km, 5 μg/ml) as described above in “Plate cultivation of M. gryphiswaldense cells for phenotypic screening.”

### Screening for M. gryphiswaldense Tn*5* insertion mutants.

After at least 14 days at 28°C, M. gryphiswaldense conjugation colonies able to grow on FSM-Km, indicating a Tn*5* insertion in the genome, were screened for Wmag and Nmag mutants by colony color. This screening was purely visual: only colonies with a color strikingly different from the dark brown color of the wild type (e.g., cream to whitish) were picked, regrown in 96-well plates, and then cultivated in Hungate tubes. At this stage, the *c_mag_* value of the mutant culture was measured, and a sample for inspection by TEM was prepared.

### Testing for spontaneous deletions in MAI.

To account for the expected high rate of spontaneous MAI rearrangements in M. gryphiswaldense (12, 29), all clones found by ARB-PCR (see below) to carry a Tn*5* insertion in genes outside the MAI were checked for deletions in the *mamAB* operon. Mutants were initially screened for the presence of each gene within the 16-kb region of the *mamAB* operon. PCR was used to amplify 1- to 3-kb sections of the *mam* gene cluster to determine their presence, absence, or change in length. Primers for this screening PCR are listed in Table S1B.

### Identification of Tn*5* interrupted genes by mapping of Tn insertion sites.

Mutants with apparently intact MAI were selected, and transposon insertion sites were identified by arbitrary PCR (ARB-PCR) (41, 71) or by Cartesian pooling (72) in combination with hybrid capture (73). Transposon/genome junctions were sequenced and compared against the genomic DNA sequence of M. gryphiswaldense (GenBank accession CP027527 [locus tag MSR1L] [74]; the locus tag MGMSRv2 refers to an older genome sequence [75] with GenBank accession HG794546.1) using the BLAST algorithm to pinpoint Tn*5*-interrupted genes. All basic bioinformatic operations for genome navigation, insertion site mapping, and gene function prediction were performed in Geneious v9 (Biomatters, Ltd., Auckland, New Zealand). For graphic visualization of Tn*5* insertion site distribution across the M. gryphiswaldense genome, DNAplotter (76) was used together with the Artemis platform (77).

### Phenotypic characterization of Tn*5* mutants.

Growth of Tn*5* insertants was assessed by measuring optical density (OD) at 565 nm. Cultures with no severe growth defect were further screened for magnetic phenotype by determination of *c_mag_* (36). For a set of mutants, cells were also assessed by optical microscopy for their swimming behavior, cell shape, and alignment in response to an externally applied magnetic field. Magnetosome morphology was analyzed with respect to size, shape, and numbers per cell by TEM analysis. For this, concentrated cells were adsorbed onto carbon-coated copper grids (Science Services, Munich, Germany) and imaged at 80 kV without negative staining in a TECNAI F20 microscope (FEI, Eindhoven, Netherlands). Ultrastructural analysis of mutants provided information on modifications in magnetosome biosynthesis as well as magnetosome organization.

### Construction of vectors for markerless deletion mutagenesis.

Markerless in-frame deletion mutants were constructed using a RecA-mediated homologous recombination system as described previously (78). For the generation of the deletion plasmid, homologous regions of around 900 to 1,000 bp up- and downstream of the gene of interest were amplified with Phusion DNA polymerase (Thermo Scientific), fused by an overlapping PCR, and ligated to “blunt ends” of an EcoRV-digested pORFM-GalK vector. The deletion plasmid was transferred to M. gryphiswaldense by conjugation, using E. coli WM3064 as a donor strain. Selection for insertion mutants was conducted by incubation on solid Km-medium. After *galK*-based counterselection, correct deletion was verified by PCR and sequencing.

### (i) Gene ontology term enrichment analysis.

Protein-coding regions of the M. gryphiswaldense genome were annotated with the Blast2GO annotation workflow (79) using NCBI’s RefSeq protein databases in combination with EBI’s InterproScan service as described in the Blast2GO manual. An enrichment analysis of GO terms (Fisher’s exact test [FET]) was performed for the gene set of Tn*5* hits outside MAI leading to a stable Wmag phenotype (test list, 75 genes) against the annotated GO terms of the M. gryphiswaldense protein coding regions (reference list, 3,717 protein coding regions with 123 MAI genes excluded; GenBank accession CP027527) to test whether certain functions are overrepresented in the set of Tn*5*-interrupted genes compared to that in the genomic background (see cartoon in Fig. S1).
