# Supplementary figures and images for: Genome-Wide Identification of Essential and Auxiliary Gene Sets for Magnetosome Biosynthesis in Magnetospirillum gryphiswaldense
Source: mSystems. 2020 Nov 17;5(6):e00565-20. doi: 10.1128/mSystems.00565-20 (PMC7676999; doi:10.1128/mSystems.00565-20)

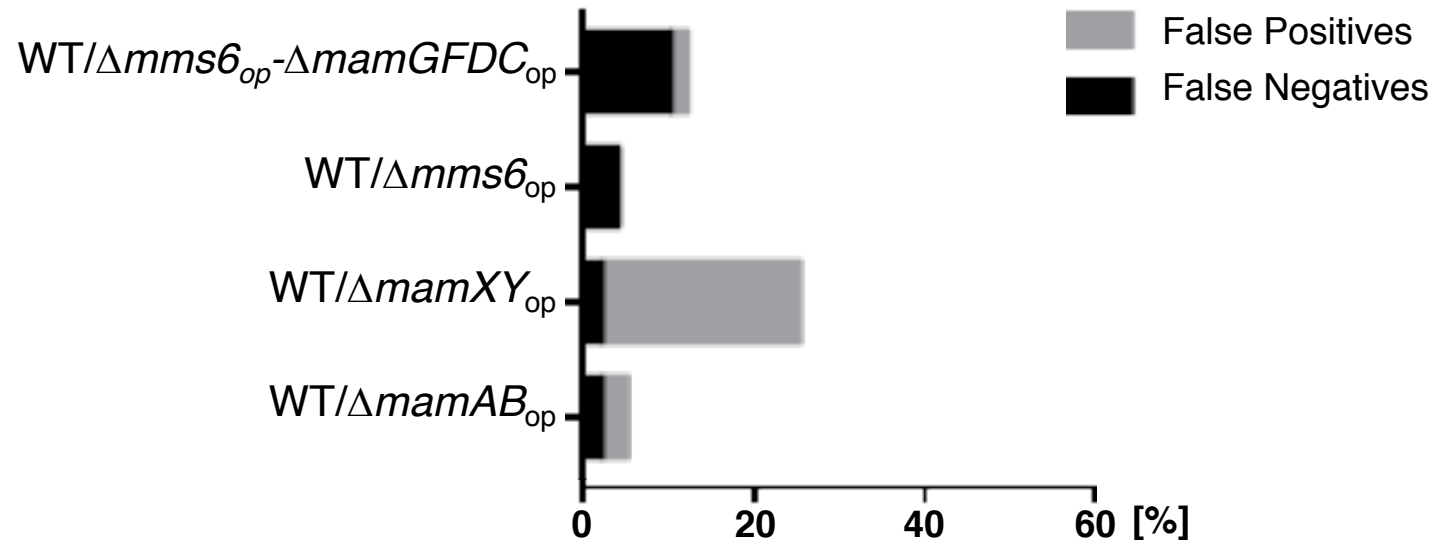

Supplement: FIG S2 [file mSystems.00565-20-sf002.pdf]

## GO term sets

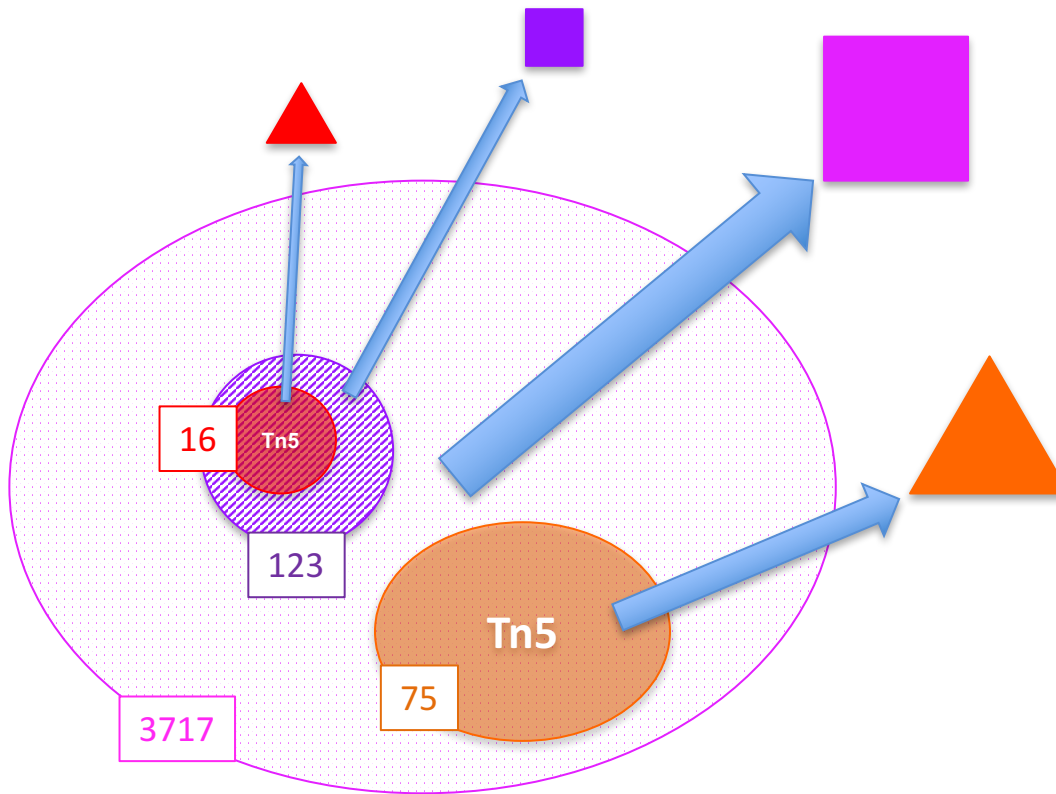

## Comparison of GO term sets

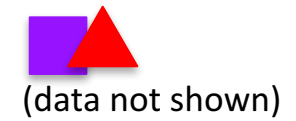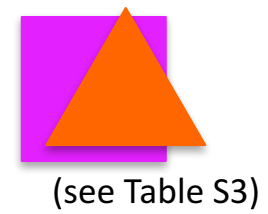

Supplement: FIG S1 [file mSystems.00565-20-sf001.pdf]

A

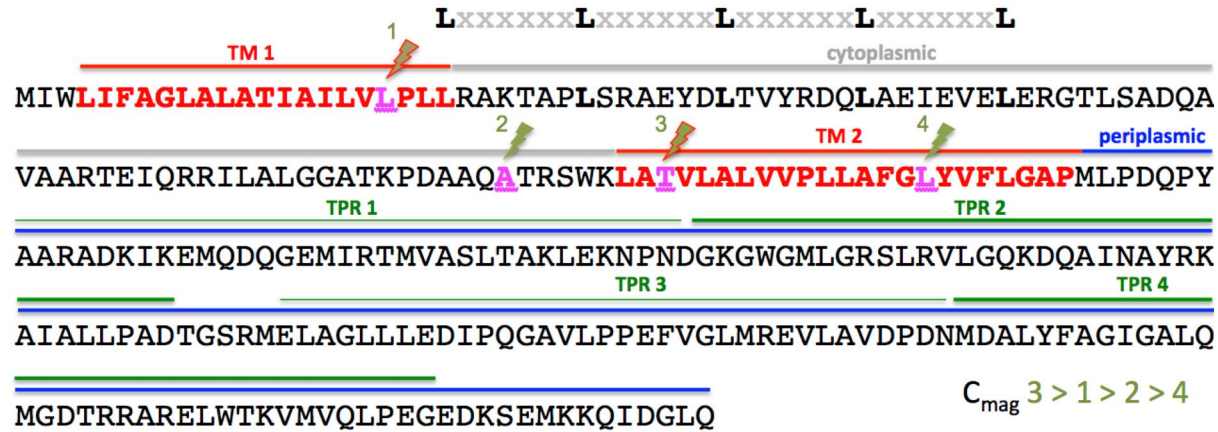

B

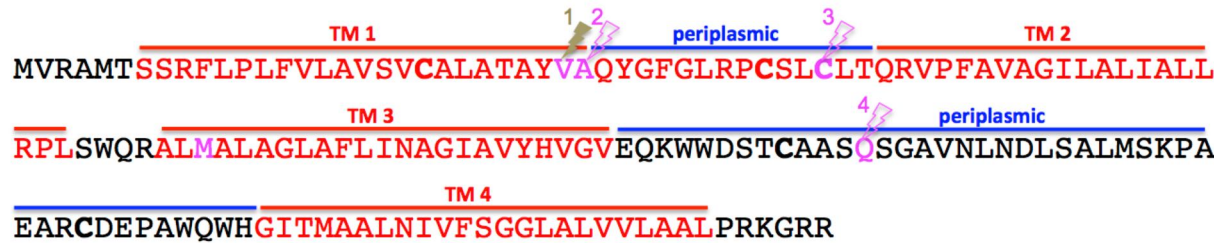

C

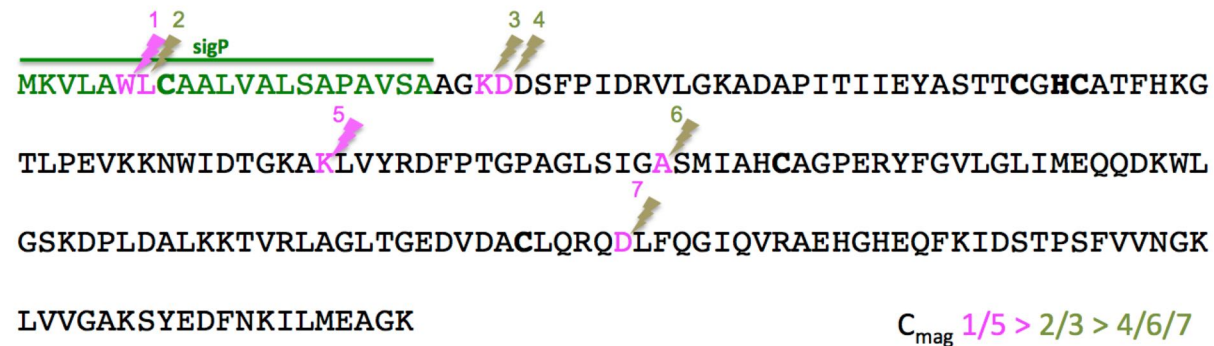

Supplement: FIG S5 [file mSystems.00565-20-sf005.pdf]
