# Supplementary material for: Genome-Wide Identification of Essential and Auxiliary Gene Sets for Magnetosome Biosynthesis in Magnetospirillum gryphiswaldense
Source: mSystems. 2020 Nov 17;5(6):e00565-20. doi: 10.1128/mSystems.00565-20 (PMC7676999; doi:10.1128/mSystems.00565-20)
Supplement: FIG S3 [file mSystems.00565-20-sf003.pdf]

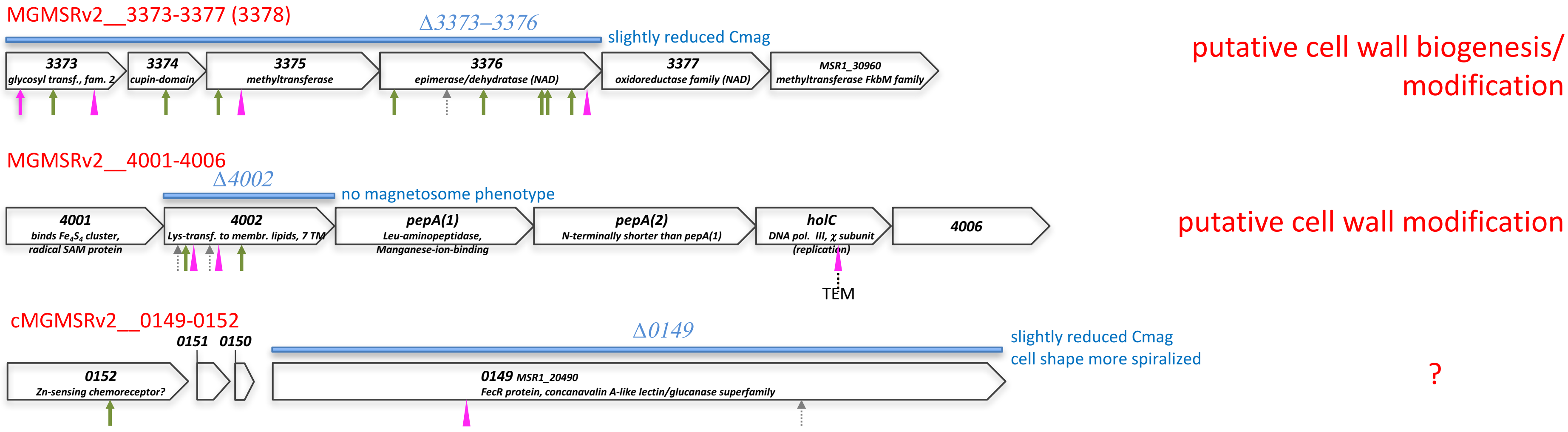
MGMSRv2__3373-3377 (3378)
Δ3373–3376
slightly reduced Cmag
3373 glycosyl transf., fam. 2
3374 cupin-domain
3375 methyltransferase
3376 epimerase/dehydratase (NAD)
3377 oxidoreductase family (NAD)
MSR1_30960 methyltransferase FkbM family
putative cell wall biogenesis/ modification
MGMSRv2__4001-4006
Δ4002
no magnetosome phenotype
4001 binds Fe_4S_4 cluster, radical SAM protein
4002 Lys-transf. to membr. lipids, 7 TM
pepA(1) Leu-aminopeptidase, Manganese-ion-binding
pepA(2) N-terminally shorter than pepA(1)
holC DNA pol. III, χ subunit (replication)
4006
TEM
putative cell wall modification
cMGMSRv2__0149-0152
0151 0150
Δ0149
slightly reduced Cmag
cell shape more spiralized
0152 Zn-sensing chemoreceptor?
0149 MSR1_20490 FecR protein, concanavalin A-like lectin/glucanase superfamily
?
