# Supplementary material for: Genome-Wide Identification of Essential and Auxiliary Gene Sets for Magnetosome Biosynthesis in Magnetospirillum gryphiswaldense
Source: mSystems. 2020 Nov 17;5(6):e00565-20. doi: 10.1128/mSystems.00565-20 (PMC7676999; doi:10.1128/mSystems.00565-20)
Supplement: TABLE S2 [file mSystems.00565-20-st002.pdf]

Table S2.A. Tn5 insertion clones selected as Nmag/Wmag during visual screening of the mutagenesis library, and characterized in detail.

exMAI	Locus-tag MSR1(L), GenBank acc. CP027526, CP027527 (R. Uebe *et al*, Genome Announc 6, 2018)	Insertion position MSR1(L)	Locus-tag MGMSRv2, GenBank acc. HG794546 (X. Wang *et al.*, Genome Announc 2, 2014)	Insertion Position in MGMSRv2	inMAI = n, exMAI = y	(putative) functional category	Annotation	Growth	Magnetic response	Growth2	Magnetic response2	Comments
**exMAI non-magnetic**	MSR1L_12470	1.235.408	MGMSRv2_1015	1.061.482	n	**peptide hydrolysis**	conserved protein of unknown function, peptidase S1 family	0,086	0	++	-	
	MSR1L_11940	1.182.493	MGMSRv2_1068	1.114.215	n	**nitrogen metabolism**	putative glutamate synthase [NADPH] small chain	0,07	0	++	-	
	MSR1L_10000	989.365	MGMSRv2_1257	?	n	**fatty acid biosynthesis**	fatty acid biosynthesis gene (beta-ketoacyl-synthase, N-terminal domain), part of a large cluster of putative fatty acid biosynthesis genes	0,14	0	+++	-	
	MSR1L_02290	232.226	MGMSRv2_2009	2.115.735	n	**signaling, MCP**	methyl-accepting chemotaxis receptor/sensory transducer	0,21	0	+++	-	
	MSR1L_01960	191.436	MGMSRv2_2042	2.156.058	n	**membrane transport?**	uncharacterized membrane protein with 2 EamA domains	0,196	0		-	
	MSR1L_32750	3.463.440	MGMSRv2_3565	3.646.533	n	**nucleotide-hydrolysis?**	putative HAD-superfamiliy hydrolase (phosphohydrolase)	0,11	0	+++	-	
	MSR1L_27630	2.940.618	MGMSRv2_3633	3.718.703	n	**signaling**	GGDEF domain-containing protein	0,13	0	+++	-	
	MSR1L_27640	2.941.097	MGMSRv2_3634	3.719.855	n	**central metabolism**	NAD(P)-dependent malic enzyme	0,077	0	++	-	
	MSR1L_23600	2.526.558	MGMSRv2_4041	4.134.803	n	**PHB biosynthesis**	poly-beta-hydroxybutyrate polymerase PhbC	0,132	0	++	-	
	MSR1L_38790	4.044.656	MGMSRv2_4153	4.251.079	n	**extracytoplasmic solute receptor**	putative TRAP-type uncharacterized transporter solute receptor, TAXI family	0,091	0	++	-	
	MSR1L_20490_corr	2.154.630	MGMSRv2_0149	170.891	n	**iron sensor?**	Laminin G-domain containing FecR-protein, Ca-binding metalloprotease	0,105	0,29	++	+	huge protein (31 kDa); periplasmic iron dicitrate sensor?
	MSR1L_18220	1.921.916	MGMSRv2_0391	419.789	n	**signaling, MCP**	methyl-accepting chemotaxis receptor	0,11	0,47	++	+	
	MSR1L_06710	669.779	MGMSRv2_0611	659.012	n	**nitrogen metabolism**	putative asparagine synthetase (glutamine-hydrolyzing)	0,121	0,36	++	+	
	MSR1L_14910	1.507.490	MGMSRv2_1555	1.600.378	n	**redox process**	electron transfer flavoprotein (alpha subunit)	0,11	0,3	+++	+	
	MSR1L_15190	1.540.079	MGMSRv2_1585	1.636.230	n	**membrane transport?**	membrane protein (DUF 2339)	0,166	0,4	++	+	large protein with 26 transmembrane domains
	MSR1L_05990	592.468	MGMSRv2_1716	1.813.723	n	**anaerobic respiration**	nitric oxide reductase (cyt *c*) subunit B (NorB)	0,024	0,3	+	+	
	MSR1L_05990	592.296	MGMSRv2_1716	1.813.903	n	**anaerobic respiration**	nitric oxide reductase (cyt *c*) subunit B (NorB)	0,14	0,07	+	+	
	MSR1L_05930	586.731	MGMSRv2_1722	1.818.698	n	**iron sensor?**	Cadherin-like domain containing FecR-proten, Ca-binding metalloprotease	0,072	0,38	++	+	
	MSR1L_20710	2.203.220	MGMSRv2_2075	2.190.040	n	**protein-protein interaction**	TPR-domain containing protein	0,1	0,58	+++	+	
	MSR1L_22700	?	MGMSRv2_2273	2.403.749	n	**periplasmatic oxidative protein folding**	thiol:disulfide interchange protein, DsbA-like	0,155	0,1	++	+	
	MSR1L_22700	?	MGMSRv2_2273	2.403.925	n	**periplasmatic oxidative protein folding**	thiol:disulfide interchange protein, DsbA-like	0,204	0,1	+++	+	

	MSR1L_22700	?	MGMSRv2_2273	2.404.129	n	**periplasmatic oxidative protein folding**	thiol:disulfide interchange protein, DsbA-like	0,076	0,24	++	+	
	MSR1L_38410	4.012.732	MGMSRv2_2474	2.585.698	n	**DNA repair**	DNA mismatch repair protein MutL	0,18	0,78	+++	+	
	MSR1L_27560	2.931.775	MGMSRv2_3625	3.709.861	n	**flagellar biosynthesis**	flagellar biosynthesis protein FlhA	0,22	0,22	+++	+	
	MSR1L_25430	2.703.940	MGMSRv2_3871	3.964.826	n	**oligopeptide transport?**	putative oligopeptide transporter subunit, periplasmic-binding component of ABC superfamily transporter	0,106	0,36	++	+	
	MSR1L_00450	50.460	MGMSRv2_0031	?	n	**signaling, histidine kinase**	two-component sensor histidine kinase	0,16	0,8	+++	++	
	MSR1L_01450	139.796	MGMSRv2_0134	127.051	n	**?**	uncharacterized protein	0,15	1	+++	++	intergenic (upstream of gene)
	MSR1L_20000	2.092.038	MGMSRv2_0199	255.584	n	**membrane transport**	inner-membrane translocator, LivM-like	0,15	0,67	+++	++	
	MSR1L_19740	2.059.355	MGMSRv2_0225	268.207	n	**?**	probable intracellular septation protein A	0,125	0	++	++	gene *ispZ;* absence of protein can cause a cell division defect in an intracellularly replicating bacterium
	MSR1L_19360	2.023.169	MGMSRv2_0266	305.286	n	**?**	Cadherin-like domain containing proten, Ca-binding metalloprotease	0,244	0,86	+++	++	
	MSR1L_30250	3.200.894	MGMSRv2_0365	399.091	n	**solute transport**	ammonium transporter AmtB	0,16	1,25	+++	++	
	MSR1L_18150	1.909.781	MGMSRv2_0398	429.605	n	**nutrient mobilization**	exopolyphosphatase	0,12	1,44	+++	++	
	MSR1L_17640	1.847.099	MGMSRv2_0450	492.828	n	**redox process**	FixG-like cyt *c* oxidase (iron-sulfur cluster -binding protein)	0,21	0,78	+++	++	
	MSR1L_17440	1.829.717	MGMSRv2_0469	509.958	n	**sulfur metabolism**	phosphoadenylyl-sulfate reductase (thioredoxin) CysH	0,081	1,05	++	++	
	MSR1L_17430	1.828.697	MGMSRv2_0470	510.978	n	**sulfur metabolism**	sulfate adenylyltransferase CysD	0,13	0,41	+++	++	
	MSR1L_17040	1.784.492	MGMSRv2_0511	556.222	n	**periplasmatic oxidative protein folding**	putative disulfide bond formation protein DsbB	0,13	0,31	+++	++	
	MSR1L_16850	1.763.821	MGMSRv2_0532	577.258	n	**?**	DU155 domain-containing protein	0,14	0,87	+++	++	
	MSR1L_16320	1.705.544	MGMSRv2_0732	767.537	n	**signaling, histidine kinase**	sensor histidine kinase with N-terminal methylesterase/transferase domains	0,078	0,87	++	++	
	MSR1L_16160	1.686.013	MGMSRv2_0749	785.805	n	**transcription regulation**	lambda repressor-like helix-turn-helix domain protein	0,158	0,72	++	++	
	MSR1L_14090	1.409.995	MGMSRv2_0853	897.589	n	**glycan biosynthesis**	glycogen synthase GlgA	0,16	1,15	+++	++	
	MSR1L_13060/MSR1L_13070	1.298.733	MGMSRv2_0953/MGMSRv2_0952	996.588	n	**transcription regulation**	transcriptional regulator, TetR family	0,13	1,36	+++	++	adjacent to *mipZ* , but divergently transcribed
	MSR1L_11790	1.168.928	MGMSRv2_1082	1.129.020	n	**stringent response**	bifunctional (P)ppGpp synthetase II and guanosine-3',5'-bis pyrophosphate 3'-pyrophosphohydrolase, RelA/SpoT family	0,101	0,74	++	++	
	MSR1L_11550	1.142.149	MGMSRv2_1113	1.157.041	n	**signaling, histidine kinase**	sensor histidine kinase	0,088	0,49	+++	++	
	MSR1L_11350	1.121.622	MGMSRv2_1133	1.178.778	n	**?**	beta-lactamase superfamily protein	0,13	1,37	+++	++	
	MSR1L_10260	1.014.414	MGMSRv2_1231	1.277.279	n	**signaling, phosphorelay**	two component sensor histidine kinase/response regulator hybrid protein with phosphorelay domain	0,11	0,88	+++	++	
	MSR1L_10050	991.939	MGMSRv2_1252	1.300.432	n	**?**	putative AMP-dependent synthetase and ligase	0,13	1,29	+++	++	

**exMAI weakly magnetic**	MSR1L_09800	968.014	MGMSRv2_1279	1.325.408	n	**isopreonoid biosynthesis**	geranyltranstransferase IspA	0,091	1	++	++	
	MSR1L_09720	961.143	MGMSRv2_1295	1.338.003	n	**redox process**	NAD(FAD)-dependent disulfide oxidoreductase	0,11	0,94	+++	++	
	MSR1L_08390	832.930	MGMSRv2_1403	1.444.705	n	**anaerobic respiration**	nitrite reductase NirS (cyt *cd1*)	0,05	0,84	++	++	
	MSR1L_08250	818.664	MGMSRv2_1417	1.459.651	n	**outer membrane structure**	OmpA-like domain-containing protein	0,069	0,34	++	++	
	MSR1L_15000	1.516.671	MGMSRv2_1565	1.609.691	n	**solute transport?**	uncharacterized membrane protein with 1 EamA domain	0,11	1,72	+++	++	
	MSR1L_15530	1.578.098	MGMSRv2_1617	1.669.557	n	**redox process**	putative short chain acyl-CoA dehydrogenase	0,13	1,11	+++	++	
	MSR1L_05880	580.065	MGMSRv2_1726	1.826.130	n	**?**	uncharacterized membrane protein	0,1	0,94	+++	++	intergenic region 3684/3683 (upstream of gene)
	MSR1L_05370	529.524	MGMSRv2_1777	1.876.671	n	**signaling, histidine kinase**	two-component sensor histidine kinase	0,11	0,82	+++	++	
	MSR1L_05070	495.250	MGMSRv2_1810	1.910.567	n	**lactate utilization?**	LUD-domain containing protein	0,05	0,97	+	++	
	MSR1L_26390	2.794.492	MGMSRv2_1971	2.068.843	n	**?**	uncharacterized protein	0,1	1,54	+++	++	
	MSR1L_02270	227.319	MGMSRv2_2011	2.120.642	n	**ammonium assimilation**	glutamate synthase [NADPH] large chain	0,14	0,87	+++	++	
	MSR1L_02170	211.717	MGMSRv2_2021	2.136.272	n	**membrane transport, nitrogen metabolism**	putative ABC transporter, urea, permease protein UrtE	0,184	0,69	+++	++	
	MSR1L_21340	2.276.260	MGMSRv2_2139	2.263.264	n	**cofactor biosynthesis**	cobalamin biosynthesis protein, CobW/P47K family protein	0,08	1,06	+++	++	
	MSR1L_22700	2.419.586	MGMSRv2_2273	2.404.131	n	**periplasmatic oxidative protein folding**	thiol:disulfide interchange protein, DsbA-like	0,075	0,63	++	++	
	MSR1L_22700	2.419.644	MGMSRv2_2273	2.404.182	n	**periplasmatic oxidative protein folding**	thiol:disulfide interchange protein, DsbA-like	0,055	0,98	++	++	
	MSR1L_06440	640.122	MGMSRv2_2461	2.576.127	n	**?**	uncharacterized exported protein	0,2	1,41	+++	++	
	MSR1L_38310	4.006.480	MGMSRv2_2484	2.591.828	n	**?**	uncharacterized protein	0,17	0,43	+++	++	intergenic (upstream of gene); downstream of bacterio-hemerythrin
	MSR1L_37510	3.931.308	MGMSRv2_2540	2.648.340	n	**?**	bacteriophage capsid protein	0,135	0,8	+++	++	region encoding phage proteins!
	MSR1L_36930	3.876.892	MGMSRv2_2673	2.772.479	n	**carbon metabolism**	methylmalonyl-CoA mutase	0,09	1,25	++	++	
	MSR1L_36480	3.835.835	MGMSRv2_2712	2.811.816	n	**protein degradation**	metalloprotease TldD/PmbA (zinc-dependent)	0,12	1,3	+++	++	antibiotic maturation/secretion? CcdA (cyt *c* biogenesis protein) degradation?
	MSR1L_36430	?	MGMSRv2_2717	2.817.087	n	**iron sensor?**	FecR-protein, Ca-binding metalloprotease	0,148	0,88	+++	++	
	MSR1L_35320	3.707.072	MGMSRv2_2825	2.934.877	n	**?**	putative SAM-dependent methyltransferase	0,1	0,42	+++	++	
	MSR1L_34190	3.604.410	MGMSRv2_2950	3.045.740	n	**redox process, nitrogen metabolism**	nitrogen fixation protein FixG	0,155	0,92	++	++	
	MSR1L_33940	3.583.056	MGMSRv2_2976	3.068.552	n	**flagellum**	flagellar biosynthesis protein FliP	0,142	0,7	+++	++	
	MSR1L_33570	3.547.505	MGMSRv2_3009	3.101.013	n	**?**	exported protein with 2 DUF1566	0,101	0,79	++	++	
	MSR1L_28800	3.052.140	MGMSRv2_3151	3.229.708	n	**fatty acid metabolism**	putative acyl-CoA carboxylase biotin-carrying subunit alpha chain AccC	0,2	0,9	+++	++	
	MSR1L_29190	3.089.592	MGMSRv2_3192	3.268.233	n	**redox process**	cytochrome *c* -type biogenesis protein CcmI (CycH)	0,08	0,96	+++	++	

	MSR1L_29190	3.089.617	MGMSRv2_3192	3.268.257	n	**redox process**	cytochrome *c* -type biogenesis protein CcmI (CycH)	0,23	0,96	+++	++	
	MSR1L_29190	3.089.338	MGMSRv2_3192	3.268.033	n	**redox process**	cytochrome *c* -type biogenesis protein CcmI (CycH)	0,12	1,16	++	++	
	MSR1L_29190	?	MGMSRv2_3192	3.268.294	n	**redox process**	cytochrome *c* -type biogenesis protein CcmI (CycH)	0,173	0,79	+++	++	
	MSR1L_29810	3.161.209	MGMSRv2_3255	3.337.096	n	**carbon metabolism**	methylmalonyl-CoA mutase	0,09	0,9	+++	++	
	MSR1L_30910	3.266.440	MGMSRv2_3373	3.444.257	n	**cell wall formation**	putative glycosyltransferase, family 2	0,101	0,79	++	++	
	MSR1L_30910	?	MGMSRv2_3373	3.444.827	n	**cell wall formation**	putative glycosyltransferase, family 2	0,112	0,7	++	++	
	MSR1L_30930	3.267.623	MGMSRv2_3375	3.445.620	n	**?**	exported protein, SAM-dependent methyltransferase	0,13	1	++	++	
	MSR1L_30940	?	MGMSRv2_3376	3.447.717	n	**cell wall formation**	putative NAD-dependent epimerase/dehydratase	0,19	0,77	+++	++	
	MSR1L_32520	3.437.807	MGMSRv2_3542	3.620.060	n	**cell wall formation**	capsular polysaccharide biosynthesis protein CapD	0,11	0,51	+++	++	
	MSR1L_32700	3.457.519	MGMSRv2_3560	3.640.612	n	**methionine biosynthesis, sulfur metabolism**	O-succinylhomoserine sufhydrylase MetZ	0,15	1,07	+++	++	
	MSR1L_27180	2.889.518	MGMSRv2_3585	3.666.512	n	**signaling, MCP**	methyl-accepting chemotaxis receptor/sensory transducer	0,24	0,94	+++	++	
	MSR1L_27780	2.957.189	MGMSRv2_3648	3.735.057	n	**signaling, phosphorelay**	Hpt domain-containing protein	0,11	0,95	+++	++	
	MSR1L_26700	2.832.812	MGMSRv2_3736	3.826.289	n	**anaerobic cofactor biosynthesis**	putative sirohydrochlorin cobaltochelatase CbiX	0,113	1,11	+++	++	
	MSR1L_24710	2.633.560	MGMSRv2_3942	4.034.134	n	**redox process, fatty acid metabolism**	3-hydroxyacyl-CoA dehydrogenase	0,19	0,94	+++	++	
	MSR1L_24490/MSR1L_24500	2.611.886	MGMSRv2_3967/3966	4.056.678	n	**?**	putative cytokinin riboside 5'-monophosphate phosphoribohydrolase (LOG family protein) / beta-lactamase superfamily protein	0,13	1,12	+++	++	may affect 2 genes: hit in intergenic region (upstream of 2 divergently transcribed genes)
	MSR1L_24400	2.605.496	MGMSRv2_3976	4.063.068	n	**?**	putative arsenite methyltransferase	0,11	1,12	+++	++	
	MSR1L_24330	2.600.607	MGMSRv2_3985	4.069.370	n	**?**	uncharacterized exported protein	0,135	0,91	++	++	
	MSR1L_24180	?	MGMSRv2_4002	4.089.224	n	**membrane lipid modification?**	conserved membrane protein of unknown function, phosphatidylglycerol lysyltransferase?	0,122	0,93	++	++	
	MSR1L_24180	2.581.355	MGMSRv2_4002	4.089.325	n	**membrane lipid modification?**	conserved membrane protein of unknown function, phosphatidylglycerol lysyltransferase	0,077	0,6	++	++	
	MSR1L_24150	?	MGMSRv2_4005	4.093.116	n	**DNA replication**	DNA polymerase III subunit chi	0,15	0,53	+++	++	
	MSR1L_38770	4.042.345	MGMSRv2_4151	4.247.156	n	**signaling**	conserved protien of unknown function-diguanylate cyclase (GGDEF and EAL domains)	0,086	0,7	++	++	
	MSR1L_39790	4.154.613	MGMSRv2_4260	4.364.696	n	**?**	conserved protein of unknown function (TPR repeat)	0,15	1,04	+++	++	
	MSR1L_20470	2.151.586	MGMSRv2_0152	175.870	n	**signaling, MCP**	methyl-accepting chemotaxis receptor, zinc-binding	0,119	1,18	++	+++	
	MSR1L_18630	1.950.998	MGMSRv2_0352	388.660	n	**?**	SIR2_2 domain-containing protein	0,122	1,28	+++	+++	

MSR1L_18100	1.902.386	MGMSRv2_0404	437.853	n	**signaling, MCP**	methyl-accepting chemotaxis receptor with hemerythrin-like metal-binding domain	0,22	1,39	+++	+++	
MSR1L_18080	1.898.487	MGMSRv2_0406	439.802	n	**fatty acid biosynthesis**	acetyl-CoA carboxylase, biotin carboxylase subunit	0,152	1,11	++	+++	
MSR1L_17950	1.883.271	MGMSRv2_0419	456.515	n	**transcription regulation**	putative transcriptional regulator, MarR-type	0,243	1,36	+++	+++	
MSR1L_17450	1.831.777	MGMSRv2_0468	508.119	n	**sulfur metabolism**	putative sulfite reductase	0,15	1,57	+++	+++	
MSR1L_17440	1.829.666	MGMSRv2_0469	509.963	n	**sulfate assimilation**	phosphoadenylyl-sulfate reductase (thioredoxin) CysH	0,1	1,73	+++	+++	
MSR1L_17430	1.828.762	MGMSRv2_0470	510.913	n	**sulfate assimilation**	sulfate adenylyltransferase CysD	0,11	1,72	+++	+++	
MSR1L_17420	1.827.992	MGMSRv2_0471	511.608	n	**sulfate assimilation**	adenylylsulfate kinase CysC	0,18	1,23	+++	+++	
MSR1L_17420	1.828.192	MGMSRv2_0471	512.275	n	**sulfate assimilation**	adenylylsulfate kinase CysC	0,107	1,28	+++	+++	
MSR1L_16570	1.732.497	MGMSRv2_0560	609.876	n	**transport**	phosphate transport system permease protein PstA	0,071	0	++	+++	
MSR1L_06670	664.301	MGMSRv2_0607	653.093	n	**exo/lipopolysaccharide biosynthesis**	putative succinoglycan biosynthesis transport protein ExoP	0,146	1,11	+++	+++	intergenic region only in GenBank acc. CP027526, CP027527 (upstream of gene)
MSR1L_06690	667.630	MGMSRv2_0609	655.028	n	**exo/lipopolysaccharide biosynthesis**	membrane protein, putative O-antigen polymerase	0,1	1,01	+++	+++	
MSR1L_12860	1.276.617	MGMSRv2_0973	1.018.940	n	**lipid binding/transfer**	coenzyme Q-binding protein COQ10, START domain-containing protein	0,135	1,46	++	+++	
MSR1L_12410	1.226.809	MGMSRv2_1021	1.069.130	n	**?**	cyclic nucleotide-binding domain-containing protein	0,203	1,32	+++	+++	
MSR1L_11790	1.167.339	MGMSRv2_1082	1.129.502	n	**stringent response**	bifunctional (P)ppGpp synthetase II and guanosine-3',5'-bis pyrophosphate 3'-pyrophosphohydrolase, RelA/SpoT family	0,17	1,62	+++	+++	
MSR1L_11470	1.142.149	MGMSRv2_1121	1.168.412	n	**?**	uncharacterized protein	0,24	1,47	+++	+++	
MSR1L_10730	?	MGMSRv2_1186	1.232.186	n	**type I protein secretion**	type 1 export protein	0,163	1,23	++	+++	
MSR1L_10730	1.059.011	MGMSRv2_1186	1.232.549	n	**type I protein secretion**	type 1 export protein	0,104	1,19	++	+++	
MSR1L_10070	993.224	MGMSRv2_1250	1.298.595	n	**cell wall formation**	lipid A biosynthesis acyltransferase	0,164	1,45	++	+++	
MSR1L_09470/09480	937.138	MGMSRv2_1319/1320	1.362.228	n	**redox process, respiratory chain**	NADH-quinone oxidoreductase subunit I (NuoI)	0,14	1,15	+++	+++	intergenic region 1319/1320 (upstream of gene 1319, *nuoI*)
MSR1L_05750	569.512	MGMSRv2_1739	1.837.060	n	**transport**	ABC-transporter, ATP binding component	0,141	1,26	++	+++	
MSR1L_06450	640.814	MGMSRv2_1956	2.051.507	n	**?**	uncharacterized protein with transmembrane domain	0,142	1,06	+++	+++	
MSR1L_26450	2.801.770	MGMSRv2_1977	2.076.121	n	**transport**	putative heme/hemopexin transporter protein HxuB	0,1	1,77	+++	+++	
MSR1L_02420	246.061	MGMSRv2_1996	2.101.926	n	**?**	alpha/beta hydrolase family protein	0,14	1,28	+++	+++	
MSR1L_01820	177.578	MGMSRv2_2056	2.170.297	n	**signaling, histidine kinase**	sensor histidine kinase with N-terminal sodium/solute symporter domain	0,137	1,39	++	+++	
MSR1L_01720	166.193	MGMSRv2_2066	2.180.906	n	**metal-binding**	putative zinc- or iron-chelating domain containing protein	0,123	1,19	++	+++	
MSR1L_21760	2.318.242	MGMSRv2_2179	2.304.151	n	**?**	conserved protein of unknown function (TPR repeat and ferritin-like domain)	0,14	0,96	+++	+++	
MSR1L_22700	2.419.427	MGMSRv2_2273	2.403.972	n	**periplasmatic oxidative protein folding**	thiol:disulfide interchange protein, DsbA-like	0,168	1,09	+++	+++	

exMAI WTmagnetic

MSR1L_22700	2.419.656	MGMSRv2_2273	2.404.184	n	**periplasmatic oxidative protein folding**	thiol:disulfide interchange protein, DsbA-like	0,249	1,34	+++	+++	
MSR1L_38000	3.973.131	MGMSRv2_2515	2.624.981	n	**nitrogen metabolism**	nitrogen-fixation sustaining protein CowN	0,118	1,24	++	+++	
MSR1L_36200	3.800.006	MGMSRv2_2734	2.842.742	n	**oxygen defence?**	redoxin/thioredoxin domain-containing exported protein	0,202	1,58	+++	+++	
MSR1L_35190	?	MGMSRv2_2838	2.944.375	n	**signaling, histidine kinase**	two-component sensor histidine kinase	0,2	1,51	+++	+++	
MSR1L_34740	3.657.073	MGMSRv2_2882	2.983.125	n	**translation**	methionyl-tRNA formyltransferase Fmt	0,18	1,21	+++	+++	
MSR1L_34720	3.654.049	MGMSRv2_2884	2.986.036	n	**signaling, MCP**	methyl-accepting chemotaxis receptor	0,227	1,28	+++	+++	
MSR1L_34000	3.587.815	MGMSRv2_2970	3.063.271	n	**redox process, chaperone function**	heat shock protein Hsp33 family protein	0,089	2,09	++	+++	
MSR1L_33770	3.567.287	MGMSRv2_2993	3.084.607	n	**?**	uncharacterized protein with transmembrane domains	0,365	1,35	+++	+++	
MSR1L_33430	3.534.455	MGMSRv2_3048	?	n	**DNA restriction**	type I site-specific restriction-modification system, R subunit	0,15	1,16	+++	+++	
MSR1L_29370	?	MGMSRv2_3209	3.285.975	n	**DNA repair**	DNA mismatch repair protein MutS	0,233	1,44	+++	+++	
MSR1L_30580	?	MGMSRv2_3332	3.406.750	n	**?**	uncharacterized protein	0,163	1,46	++	+++	
MSR1L_30810	3.255.094	MGMSRv2_3362	3.431.344	n	**cofactor biosynthesis**	molybdenum cofactor biosynthesis protein MoaA	0,087	1,16	++	+++	
MSR1L_30910	?	MGMSRv2_3373	3.444.257	n	**cell wall formation**	putative glycosyltransferase, family 2	0,246	1,12	++	+++	
MSR1L_30910	?	MGMSRv2_3373	3.444.658	n	**cell wall formation**	putative glycosyltransferase, family 2	0,09	1,18	++	+++	
MSR1L_30920	3.267.147	MGMSRv2_3374	3.445.257	n	**?**	conserved protein of unknown function	0,146	1,05	++	+++	
MSR1L_30930	3.267.623	MGMSRv2_3375	3.445.480	n	**?**	exported protein, SAM-dependent methyltransferase	0,221	1,16	+++	+++	
MSR1L_30940	?	MGMSRv2_3376	3.446.709	n	**cell wall formation**	putative NAD-dependent epimerase/dehydratase	0,084	1,03	++	+++	
MSR1L_30940	?	MGMSRv2_3376	3.447.280	n	**cell wall formation**	putative NAD-dependent epimerase/dehydratase	0,06	1,31	++	+++	
MSR1L_30940	3.268.871	MGMSRv2_3376	3.447.520	n	**cell wall formation**	putative NAD-dependent epimerase/dehydratase	0,115	1,05	++	+++	
MSR1L_30940	?	MGMSRv2_3376	3.447.526	n	**cell wall formation**	putative NAD-dependent epimerase/dehydratase	0,211	1,21	+++	+++	
MSR1L_30940	?	MGMSRv2_3376	3.447.644	n	**cell wall formation**	putative NAD-dependent epimerase/dehydratase	0,22	1,28	+++	+++	
MSR1L_27160	2.886.228	MGMSRv2_3583	3.663.641	n	**peptide hydrolysis**	putative Xaa-Pro aminopeptidase	0,198	1,35	+++	+++	
MSR1L_27190	2.889.708	MGMSRv2_3586	3.668.956	n	**glutathione metabolism**	5-oxoprolinase OplA	0,128	1,49	++	+++	
MSR1L_26800	2.847.442	MGMSRv2_3727	3.811.056	n	**chemotaxis?**	CheR-like methyltransferase with C-terminal TPR-domain	0,131	1,13	++	+++	
MSR1L_25740	2.733.921	MGMSRv2_3840	3.936.201	n	**cell wall formation**	lipopolysaccharide assembly protein A-domain containing protein	0,15	0	+++	+++	
MSR1L_24680	2.631.081	MGMSRv2_3945	4.036.591	n	**cofactor biosynthesis**	ATP:cob(I)alamin adenosyltransferase	0,209	1,18	+++	+++	
MSR1L_24530	2.614.968	MGMSRv2_3963	4.053.246	n	**?**	conserved inner membrane protein, DUF420	0,185	1,24	+++	+++	
MSR1L_24260	2.590.538	MGMSRv2_3992	4.078.926	n	**signaling, MCP**	methyl-accepting chemotaxis receptor	0,1	1,57	+++	+++	

	MSR1L_24180	?	MGMSRv2_4002	4.089.167	n	**membrane lipid modification?**	conserved membrane protein of unknown function, phosphatidylglycerol lysyltransferase	0,118	1,06	++	+++	
	MSR1L_24180	?	MGMSRv2_4002	4.089.537	n	**membrane lipid modification?**	conserved membrane protein of unknown function, phosphatidylglycerol lysyltransferase	0,179	1,22	+++	+++	
	MSR1L_39470	4.123.757	MGMSRv2_4227	4.332.947	n	**carbon metabolism, gluconeogenesis**	pyruvate phosphate dikinase PpdK	0,1	1,13	++	+++	
	MSR1L_39620	4.136.506	MGMSRv2_4243	4.346.854	n	**signaling, histidine kinase**	sensor histidine kinase	0,233	1,21	+++	+++	
	MSR1L_26530	2.811.416	MGMSRv2_5S_rRNA_2	2.085.763	n	**ribosome component**	5S_rRNA_2	0,14	1,13	+++	+++	
	MSR1L_02570	262.088	MGMSRv2_5S_rRNA_2	?	n	**ribosome component**	5S_rRNA_2	0,14	1,13	+++	+++	

inMAI	Locus-tag MSR1(L), GenBank acc. CP027526, CP027527 (4)	Insertion position MSR1(L)	Locus-tag MGMSRv2, GenBank acc. HG794546 (5)	Insertion Position in MGMSRv2	inMAI = n, exMAI = y	(putative) functional category	Annotation	Growth	Magnetic response	Growth2	Magnetic response2	comments
	MSR1L_03470	335.203	MGMSRv2_2368	2.489.301	y		MamB	0,205	0	+++	-	
	MSR1L_03420	330.858	MGMSRv2_2373	2.493.753	y		MamO	0,103	0,04	++	-	
	MSR1L_03380	327.097	MGMSRv2_2377	2.497.515	y		MamK	0,103	0,04	++	-	
	MSR1L_03360	?	MGMSRv2_2379	2.500.968	y		MamE	0,224	0	+++	-	
	MSR1L_03470	?	MGMSRv2_2368	?	y		MamB	0,176	0	+++	-	
	MSR1L_03470	?	MGMSRv2_2368	?	y		MamB	0,181	0	+++	-	
	MSR1L_03390	327.647	MGMSRv2_2377	?	y		MamL	0,261	0	+++	-	
	MSR1L_03380	326.862	MGMSRv2_2377	2.479.749	y		MamK	0,104	0,03	++	-	
	MSR1L_03470	?	MGMSRv2_2368	2.489.212	y		MamB	0,146	0	++	-	
	MSR1L_03470	?	MGMSRv2_2368	2.489.586	y		MamB	0,125	0	++	-	
	MSR1L_03470	334.986	MGMSRv2_2368	2.489.609	y		MamB	0,162	0	++	-	
	MSR1L_03450	?	MGMSRv2_2370	2.490.149	y		MamQ	0,199	0	+++	-	
	MSR1L_03450	?	MGMSRv2_2370	2.490.387	y		MamQ	0,119	0	++	-	
	MSR1L_03450	?	MGMSRv2_2370	2.490.500	y		MamQ	0,197	0	+++	-	
	MSR1L_03450	?	MGMSRv2_2370	2.490.548	y		MamQ	0,188	0	+++	-	
	MSR1L_03440	?	MGMSRv2_2371	2.491.210	y		MamA	0,217	0	+++	-	
	MSR1L_03430	?	MGMSRv2_2372	2.492.065	y		MamP	0,177	0	+++	-	
	MSR1L_03420	?	MGMSRv2_2373	2.492.720	y		MamO	0,209	0	+++	-	
	MSR1L_03420	?	MGMSRv2_2373	2.492.799	y		MamO	0,233	0	+++	-	
	MSR1L_03420	?	MGMSRv2_2373	2.492.922	y		MamO	0,195	0	+++	-	
	MSR1L_03420	?	MGMSRv2_2373	2.493.134	y		MamO	0,195	0	+++	-	
	MSR1L_03420	330.654	MGMSRv2_2373	2.493.431	y		MamO	0,2	0	+++	-	
	MSR1L_03420	331.008	MGMSRv2_2373	2.493.584	y		MamO	0,203	0	+++	-	
	MSR1L_03420	?	MGMSRv2_2373	2.493.591	y		MamO	0,124	0	++	-	
	MSR1L_03420	330.506	MGMSRv2_2373	2.493.908	y		MamO	0,178	0	+++	-	
	MSR1L_03420	330.542	MGMSRv2_2373	2.494.052	y		MamO	0,176	0	+++	-	
	MSR1L_03410	?	MGMSRv2_2374	2.494.784	y		MamN	0,113	0	++	-	
	MSR1L_03400	328.406	MGMSRv2_2375	2.495.915	y		MamM	0,199	0	+++	-	
	MSR1L_03400	328.399	MGMSRv2_2375	2.495.915	y		MamM	0,143	0	++	-	
	MSR1L_03400	?	MGMSRv2_2375	2.495.960	y		MamM	0,116	0	++	-	
	MSR1L_03400	?	MGMSRv2_2375	2.496.081	y		MamM	0,115	0	++	-	
	MSR1L_03400	328.397	MGMSRv2_2375	2.496.206	y		MamM	0,096	0,03	++	-	
	MSR1L_03400	?	MGMSRv2_2375	2.496.274	y		MamM	0,119	0	++	-	
	MSR1L_03400	?	MGMSRv2_2375	2.496.387	y		MamM	0,065	0	++	-	

inMAI non-magnetic	MSR1L_03400	?	MGMSRv2_2375	2.496.401	y		MamM	0,014	0	+	-	
	MSR1L_03400	?	MGMSRv2_2375	2.496.620	y		MamM	0,203	0	+++	-	
	MSR1L_03400	?	MGMSRv2_2375	2.496.643	y		MamM	0,198	0	+++	-	
	MSR1L_03380	?	MGMSRv2_2378	2.499.214	y		MamJ	0,1	0,02	+++	-	
	MSR1L_03360	?	MGMSRv2_2379	2.500.017	y		MamE	0,222	0	+++	-	
	MSR1L_03360	324.627	MGMSRv2_2379	2.500.104	y		MamE	0,208	0	+++	-	
	MSR1L_03360	?	MGMSRv2_2379	2.500.835	y		MamE	0,227	0	+++	-	
	MSR1L_03360	323.338	MGMSRv2_2379	2.501.382	y		MamE	0,185	0	+++	-	
	MSR1L_03360	?	MGMSRv2_2379	2.501.635	y		MamE	0,251	0	+++	-	
	MSR1L_03360	?	MGMSRv2_2379	2.501.720	y		MamE	0,11	0	++	-	
	MSR1L_03350	?	MGMSRv2_2380	2.501.994	y		MamI	0,135	0	++	-	
	MSR1L_03180	308.180	MGMSRv2_2396	2.515.601	y		Mms6	0,071	0	++	-	
	MSR1L_03470	335.431	MGMSRv2_2368	?	y		MamB	0,12	0	+++	-	
	MSR1L_03450	334.362	MGMSRv2_2370	?	y		MamQ	0,15	0	+++	-	
	MSR1L_03440	333.456	MGMSRv2_2371	?	y		MamA	0,092	0	++	-	
	MSR1L_03440	333.129	MGMSRv2_2371	?	y		MamA	0,13	0	+++	-	
	MSR1L_03440	333.251	MGMSRv2_2371	?	y		MamA	0,11	0	+++	-	
	MSR1L_03440	333.218	MGMSRv2_2371	?	y		MamA	0,13	0	+++	-	
	MSR1L_03420	332.100	MGMSRv2_2373	?	y		MamO	0,198	0	+++	-	
	MSR1L_03420	330.506	MGMSRv2_2373	?	y		MamO	0,185	0	+++	-	
	MSR1L_03420	330.282	MGMSRv2_2373	?	y		MamO	0,13	0	+++	-	
	MSR1L_03420	330.879	MGMSRv2_2373	?	y		MamO	0,19	0	+++	-	
	MSR1L_03420	330.927	MGMSRv2_2373	?	y		MamO	0,14	0	+++	-	
	MSR1L_03410	329.389	MGMSRv2_2374	?	y		MamN	0,14	0	+++	-	
	MSR1L_03410	329.084	MGMSRv2_2374	?	y		MamN	0,13	0	+++	-	
	MSR1L_03400	328.494	MGMSRv2_2375	?	y		MamM	0,13	0	+++	-	
	MSR1L_03400	328.494	MGMSRv2_2375	?	y		MamM	0,13	0	+++	-	
	MSR1L_03400	328.211	MGMSRv2_2375	?	y		MamM	0,16	0	+++	-	
	MSR1L_03400	327.960	MGMSRv2_2375	?	y		MamM	0,14	0	+++	-	
	MSR1L_03400	328.601	MGMSRv2_2375	?	y		MamM	0,14	0	+++	-	
	MSR1L_03400	?	MGMSRv2_2375	?	y		MamM	0,14	0	+++	-	
	MSR1L_03380	326.998	MGMSRv2_2377	?	y		MamK	0,16	0	+++	-	
	MSR1L_03380	327.270	MGMSRv2_2377	?	y		MamK	0,14	0	+++	-	
	MSR1L_03380	326.953	MGMSRv2_2377	?	y		MamK	0,14	0	+++	-	
	MSR1L_03360	323.068	MGMSRv2_2379	?	y		MamE	0,176	0	+++	-	
	MSR1L_03360	323.475	MGMSRv2_2379	?	y		MamE	0,176	0	+++	-	
	MSR1L_03360	323.823	MGMSRv2_2379	?	y		MamE	0,193	0	+++	-	
	MSR1L_03360	323.688	MGMSRv2_2379	?	y		MamE	0,188	0	+++	-	
	MSR1L_03360	324.735	MGMSRv2_2379	?	y		MamE	0,15	0	+++	-	
	MSR1L_03360	322.931	MGMSRv2_2379	?	y		MamE	0,16	0	+++	-	
	MSR1L_03360	323.936	MGMSRv2_2379	?	y		MamE	0,13	0	+++	-	
	MSR1L_03360	323.088	MGMSRv2_2379	?	y		MamE	0,15	0	+++	-	
	MSR1L_03360	324.141	MGMSRv2_2379	?	y		MamE	0,2	0	+++	-	
magnetic	MSR1L_03440	333.610	MGMSRv2_2371	?	y		MamA	0,12	0,85	+++	+	
	MSR1L_03440	333.610	MGMSRv2_2371	?	y		MamA	0,14	0,89	+++	+	
	MSR1L_03380	327.473	MGMSRv2_2377	?	y		MamK	0,103	0,44	+	+	
	MSR1L_03860	368.977	MGMSRv2_2323	2.459.845	y		MamZ	0,092	0,1	++	+	
	MSR1L_03470	?	MGMSRv2_2368	2.489.408	y		MamB	0,177	0,1	+++	+	
	MSR1L_03430	?	MGMSRv2_2372	2.491.610	y	**magnetochrome**	MamP	0,151	0,07	+++	+	
	MSR1L_03420	?	MGMSRv2_2373	2.492.443	y		MamO	0,105	0,23	+++	+	
	MSR1L_03410	?	MGMSRv2_2374	2.494.624	y		MamN	0,104	0,11	+++	+	
	MSR1L_03410	?	MGMSRv2_2374	2.494.745	y		MamN	0,111	0,06	+++	+	
	MSR1L_03380	327.647	MGMSRv2_2377	2.497.478	y		MamK	0,147	0	+++	+	

**inMAI weakly m**	MSR1L_03430	332.899	MGMSRv2_2372	?	y	**magnetochrome**	MamP	0,15	0,06	+++	+	
	MSR1L_03870	369.755	MGMSRv2_2322	2.457.648	y	**magnetochrome**	MamX	0,131	1,16	++	++	
	MSR1L_03380	326.953	MGMSRv2_2377	?	y		MamK	0,14	0,87	+++	++	
	MSR1L_03160	307.382	MGMSRv2_2398	?	y		Mms36	0,103	0,72	+++	++	
	MSR1L_02670	270.213	MGMSRv2_2312	2.446.983	y		FeoB1	0,156	0,75	++	++	
	MSR1L_02660	?	MGMSRv2_2313	2.447.939	y		FeoA1	0,144	0,93	++	++	
	MSR1L_03870	?	MGMSRv2_2322	2.457.856	y	**magnetochrome**	MamX	0,204	0,96	+++	++	
	MSR1L_03460	?	MGMSRv2_2369	2.489.871	y		MamR	0,144	0,81	++	++	
	MSR1L_03350	?	MGMSRv2_2380	2.502.080	y		MamI	0,107	0,62	+++	++	
	MSR1L_03180	308.146	MGMSRv2_2396	2.515.559	y		Mms6	0,133	0,81	+++	++	
	MSR1L_03170	307.881	MGMSRv2_2397	2.515.856	y		MmsF	0,132	0,72	++	++	
	MSR1L_03470	334.986	MGMSRv2_2368	?	y		MamB	0,15	0,6	+++	++	
	MSR1L_03340	321.630	MGMSRv2_2381	?	y		MamH	0,106	0,68	++	++	
**inMAI WTmagnetic**	MSR1L_03460	?	MGMSRv2_2369	2.490.029	y		MamR	0,229	1,1	+++	+++	
	MSR1L_03430	?	MGMSRv2_2372	2.491.777	y	**magnetochrome**	MamP	0,271	1,27	+++	+++	
	MSR1L_03340	321.870	MGMSRv2_2381	?	y		MamH	0,11	1,37	+++	+++	
	MSR1L_02670	271.399	MGMSRv2_2312	2.446.529	y		FeoB1	0,137	1,01	++	+++	
	MSR1L_02660	269.922	MGMSRv2_2313	2.447.981	y		FeoA1	0,153	1,47	++	+++	
	MSR1L_03490	336.337	MGMSRv2_2366	2.488.276	y	**magnetochrome**	MamT	0,249	1,34	+++	+++	
	MSR1L_03420	?	MGMSRv2_2373	2.492.921	y		MamO	0,124	1,43	++	+++	
	MSR1L_03420	?	MGMSRv2_2373	2.493.652	y		MamO	0,295	1,43	+++	+++	
	MSR1L_03410	?	MGMSRv2_2374	2.495.482	y		MamN	0,118	1,35	++	+++	
	MSR1L_03380	?	MGMSRv2_2377	2.497.629	y		MamK	0,271	1,27	+++	+++	
	MSR1L_03360	?	MGMSRv2_2379	2.501.663	y	**magnetochrome**	MamE	0,263	1,1	+++	+++	
	MSR1L_03340	?	MGMSRv2_2381	2.502.366	y		MamH	0,135	1,03	++	+++	
	MSR1L_03340	?	MGMSRv2_2381	2.502.685	y		MamH	0,131	1,13	++	+++	
	MSR1L_03180	308.322	MGMSRv2_2396	2.515.809	y		Mms6	0,118	1,41	++	+++	
	MSR1L_02670	270.908	MGMSRv2_2312	?	y		FeoB1	0,15	1,16	+++	+++	
	MSR1L_02670	271.404	MGMSRv2_2312	?	y		FeoB1	0,249	1,34	+++	+++	
	MSR1L_03870	369.042	MGMSRv2_2322	?	y	**magnetochrome**	MamX	0,16	1,11	+++	+++	
	MSR1L_03860	368.933	MGMSRv2_2323	?	y		MamZ	0,1	1,36	+++	+++	
	MSR1L_03420	330.630	MGMSRv2_2373	?	y		MamO	0,13	1,48	+++	+++	
	MSR1L_03410	329.389	MGMSRv2_2374	?	y		MamN	0,11	1,07	+++	+++	

Table S2.B. Tn5 insertion clones selected as Nmag/Wmag during visual screening of the mutagenesis library.

Locus-tag MSR1(L), GenBank acc. CP027526, CP027527 (R. Uebe et al, Genome Announc 6, 2018)	Insertion position MSR1(L)	Locus-tag MGMSRv2, GenBank acc. HG794546 (X. Wang et al., Genome Announc 2, 2014)	Insertion Position in MGMSRv2	inMAI = n, exMAI = y	(putative) functional category	Annotation	Comments
MSR1L_00320	35.238	MGMSRv2_0021	22.170	n	RNA	RmlB-23S RNA methyltransferase	*rlmB*
MSR1L_01530	145.647	MGMSRv2_0142	133.754	n	?	exported protein of unknown function	DUF839; TAT-signal!
MSR1L_20490	?	MGMSRv2_0149	150.840	n	toxin?	conserved protein of unknown function	huge ORF (31 kb)!
MSR1L_20280	2.124.592	MGMSRv2_0173	203.045	n	cell wall?	transglutaminase-like protein	DUF2126
MSR1L_20040	2.095.720	MGMSRv2_0195	?	n	redox	aldehyde dehydrogenase	
MSR1L_19820	2.071.226	MGMSRv2_0217	256.053	n	?	conserved protein of unknown function	TPR-protein? long operon 210-222! SrfC and SrfB (virulence effectors) encoded right upstream
MSR1L_19720	2.056.697	MGMSRv2_0227	270.168	n	purine catabolism	aminotransferase class V, purine catabolism	*pucG*
MSR1L_19360	2.023.570	MGMSRv2_0266	?	n	protein secretion?	adhesive surface protein, hemeagglutinin-like?	
MSR1L_19350	2.019.808	MGMSRv2_0267	?	n	protein secretion?	outer membrane efflux protein	PF02321; 14 proteins in MSR-1
MSR1L_19350	2.019.808	MGMSRv2_0267	?	n	protein secretion?	outer membrane efflux protein	PF02321; 14 proteins in MSR-1
MSR1L_18700	1.957.720	MGMSRv2_0345	?	n	nitrogen	nitrogenase iron protein NifH	binds 4Fe-4S-cluster
MSR1L_18160	1.912.974	MGMSRv2_0397	?	n	storage	polyphosphate kinase	*ppK*
MSR1L_17970	1.885.282	MGMSRv2_0417	?	n	transporter	ABC-transporter, substrate-binding protein	Leu-binding? Leu/Ile/Val-binding?
MSR1L_17970	1.884.455	MGMSRv2_0417	?	n	transporter	ABC-transporter, substrate-binding protein	Leu-binding? Leu/Ile/Val-binding?
MSR1L_17900	1.880.358	MGMSRv2_0424	459.294	n	transporter	ABC-transporter, ATPase component	*hmuV* (hemine transporter?) *fecCD (* ABC transporter ferric iron dicitrate permease and ABC transporter ferric iron hydroxamate periplasmic binding protein encoded upstream)
MSR1L_17870	1.876.175	MGMSRv2_0427	463.722	n	transporter	TonB dependent receptor	
MSR1L_17860	1.875.950	MGMSRv2_0428	464.592	n	regulator	AraC-type DNA - binding domain containing protein	transcriptional regulator
MSR1L_17500	1.835.661	MGMSRv2_0463	?	n	signaling, His-kin/RR	His-kinase-response regulator hybrid	GAF-like + PAS + His-Kin + RR
MSR1L_17450	1.830.615	MGMSRv2_0468	508.148	n	sulfur	sulfite reductase	
MSR1L_17450	?	MGMSRv2_0468	508.665	n	sulfur	sulfite reductase	
MSR1L_17450	?	MGMSRv2_0468	509.262	n	sulfur	sulfite reductase	

	MSR1L_17440	1.829.717	MGMSRv2_0469	510.110	n	sulfur	phosphoadenylyl-sulfate reductase (thioredoxin)	*cysH*
	MSR1L_17440	?	MGMSRv2_0469	510.114	n	sulfur	phosphoadenylyl-sulfate reductase (thioredoxin)	*cysH*
	MSR1L_17430	?	MGMSRv2_0470	511.348	n	sulfur	sulfate adenylyltransferase	*cysD*
	MSR1L_17430	1.829.014	MGMSRv2_0470	?	n	sulfur	sulfate adenylyltransferase	*cysD*
	MSR1L_17420	?	MGMSRv2_0471	511.952	n	sulfur	adenylylsulphate kinase	*cysC*
	MSR1L_17420	?	MGMSRv2_0471	513.033	n	sulfur	adenylylsulphate kinase	*cysC*
	MSR1L_17420	?	MGMSRv2_0471	513.150	n	sulfur	adenylylsulphate kinase	*cysC*
	MSR1L_17410	1.824.506	MGMSRv2_0472	?	n	signaling, His-kin	His-kinase	PAS, no HAMP, His-Kin
	MSR1L_17180	1.799.009	MGMSRv2_0495	539.233	n	sulfur	cystathionine beta-lyase	
	MSR1L_17040	?	MGMSRv2_0511	556.223	n	sulfur	disulfide bond formation protein DsbB	*dsbB*
	MSR1L_17040	?	MGMSRv2_0511	556.260	n	sulfur	disulfide bond formation protein DsbB	*dsbB*
	MSR1L_17040	?	MGMSRv2_0511	556.447	n	sulfur	disulfide bond formation protein DsbB	*dsbB*
	MSR1L_17010	1.782.185	MGMSRv2_0514	557.729	n	peptidase	peptidase family M48 protein	
	MSR1L_16940	1.776.119	MGMSRv2_0521	?	n	carbohydrate catabolism	glycogen debranching enzyme (family 13 glycoside hydrolase)	*glgX*
	MSR1L_16830	1.762.256	MGMSRv2_0534	?	n	?	uncharacterized protein	2 TM
	MSR1L_16790	1.751.203	MGMSRv2_0538	?	n	lipid metabolism	biotin carboxylase	*accC*
	MSR1L_16650	1.740.946	MGMSRv2_0551	601.019	n	phosphatase?	rhodanese-like protein	
	MSR1L_06530	647.482	MGMSRv2_0592	?	n	DNA	integrase	
	MSR1L_06710	670.859	MGMSRv2_0611	?	n	amino acid biosynthesis	asparagine synthetase (glutamine-hydrolyzing)	
	MSR1L_05360	527.032	MGMSRv2_0647	?	n	DNA	transposase	IS4 family
	MSR1L_16340	1.706.551	MGMSRv2_0730	765.000	n	metabolism	succinic semialdehyde dehydrogenase	*gabD* ; 4-amino-butyrate (GABA) degradation
	MSR1L_06910	692.410	MGMSRv2_0762	?	n	?	DUF1398 protein	
	MSR1L_14490	1.455.448	MGMSRv2_0813	?	n	regulator?	cupin-domain protein	transcription factor?
	MSR1L_14380	1.438.539	MGMSRv2_0824	861.701	n	transporter	outer membrane efflux protein	
	MSR1L_13600	1.349.563	MGMSRv2_0902	?	n	signaling, His-kin	His-kinase	x, no PAS, no HAMP, His-Kin
	MSR1L_13120	1.304.145	MGMSRv2_0947	?	n	transporter	high affinity branched-chain amino acid ABC transporter, permease protein (LivM-like)	9 TM
	MSR1L_12940	1.284.321	MGMSRv2_0965	?	n	chemotaxis	methyl-accepting chemotaxis protein	?
	MSR1L_12460	1.231.707	MGMSRv2_1016	?	n	metabolism	acyl-CoA synthetase	
	MSR1L_11550	1.143.631	MGMSRv2_1113	?	n	signaling, His-kin	His-kinase	Chase, PAS, no HAMP, His-Kin
	MSR1L_10740	1.061.185	MGMSRv2_1185	?	n	protein secretion	type I secretion membrane fusion protein, HlyD family	TIGR01843, 5 proteins in MSR-1

MSR1L_10260	1.012.228	MGMSRv2_1231	?	n	signaling, His-kin/RR	His-kinase-response regulator hybrid (Hpt phosphotransfer)	
MSR1L_10100	995.869	MGMSRv2_1247	?	n	?	membrane protein	10 TM
MSR1L_10040	991.007	MGMSRv2_1253	?	n	?	membrane protein	4 TM
MSR1L_09900	979.965	MGMSRv2_1267	1.312.679	n	transporter	TauE-like transmembrane protein	7 TM
MSR1L_09710	959.268	MGMSRv2_1296	?	n	aromatic amino acid catabolism	glutathione-S-transferase family protein	
MSR1L_09700	958.261	MGMSRv2_1297	1.340.313	n	amino acid catabolism	fumarylacetoacetate (FAA) hydrolase	
MSR1L_09340	923.359	MGMSRv2_1331	1.375.903	n	peptidase	ATP-dependent ClpA-ClpP serine protease, proteolytic subunit ClpP	*Desulfovibrio magneticus* RS-1
MSR1L_09260	912.548	MGMSRv2_1339	?	n	competence, DNA-uptake?	ComEC/Rec2-related protein	transmembrane protein
MSR1L_08990	886.867	MGMSRv2_1366	?	n	chemotaxis	methyl-accepting chemotaxis protein	4HB_MCP_1 (4helix bundle sensory module)
MSR1L_08380	831.371	MGMSRv2_1404	?	n	regulator	cNMP-binding Crp-type DNA-binding protein	
MSR1L_07420	741.545	MGMSRv2_1505	?	n	?	uncharacterized protein	
MSR1L_14690	1.475.592	MGMSRv2_1531	?	n	signaling	GGDEF-domain containing protein	6 TM; 30 proteins in MSR-1; 20 proteins in MSR-1 with additional EAL domain (and in part additional signaling domains)
MSR1L_14890	1.505.532	MGMSRv2_1553	?	n	redox	acyl-CoA dehydrogenase	
MSR1L_15490	1.574.547	MGMSRv2_1613	?	n	Flp pilus-assembly?	vWFA-protein	
MSR1L_15630	?	MGMSRv2_1627	1.688.478	n	PKS	conserved protein of unknown function containing polyketide synthase/Fatty acid synthase domain	huge orf! PKS (like 1629)!
MSR1L_15650	1.611.703	MGMSRv2_1629	?	n	PKS	polyketide synthase	
MSR1L_15810	1.644.793	MGMSRv2_1645	?	n	metabolism	phosphoesterase	
MSR1L_15920	1.651.865	MGMSRv2_1656	?	n	chemotaxis	protein-glutamate methylesterase	me-esterase CheB/me-transferase CheR/PAS domain
MSR1L_16000	1.667.809	MGMSRv2_1666	?	n	signaling	signal transduction response regulator, receiver domain	no output domain
MSR1L_16010	1.668.040	MGMSRv2_1667	?	n	chemotaxis	protein-glutamate methylesterase	me-esterase CheB/me-transferase CheR/His-kin/2xRR/GerE output
MSR1L_06180	611.172	MGMSRv2_1694	1.793.463	n	transporter	TrkA-C domain protein	citrate transporter?
MSR1L_05990	592.221	MGMSRv2_1716	1.813.021	n	nitrogen	nitric oxide reductase, subunit B	*norB*
MSR1L_05980	591.755	MGMSRv2_1717	1.814.067	n	nitrogen	nitric oxide reductase, subunit C	*norC*

**exMA**	MSR1L_04990	486.009	MGMSRv2_1818	1.918.186	n	transporter	TonB-dependent receptor	TonB-copper (like OprC, NosA); also in Ca. Magnetoovum chiemensis, Magnetobacterium bavaricum, Magnetobacterium casensis
	MSR1L_04660	454.065	MGMSRv2_1852	?	n	cell wall	dTDP-glucose 4,6-dehydratase	*rfbB*
	MSR1L_04500	433.577	MGMSRv2_1868	1.973.360	n	chemotaxis	methyl-accepting chemotaxis protein	?
	MSR1L_04410	423.819	MGMSRv2_1877	1.981.612	n	chemotaxis	methyl-accepting chemotaxis protein	?
	MSR1L_04280	408.245	MGMSRv2_1890	?	n	carbohydrate catabolism	beta-glucosidase	
	MSR1L_26390	?	MGMSRv2_1971	2.068.765	n	?	hypothetical protein	
	MSR1L_02470	251.139	MGMSRv2_1991	?	n	signaling, His-kin	His-kinase	PAS, no HAMP, His-Kin
	MSR1L_02430	246.222	MGMSRv2_1995	?	n	?	uncharacterized protein	
	MSR1L_02420	245.417	MGMSRv2_1996	2.102.495	n	cofactor biosynthesis	carboxylesterase BioH	
	MSR1L_02200	215.216	MGMSRv2_2018	2.133.125	n	transporter	ABC transporter, urea, permease protein UrtB	
	MSR1L_01960	190.748	MGMSRv2_2042	?	n	transporter	transmembrane protein	2 EamA domains
	MSR1L_20760	2.207.517	MGMSRv2_2080	2.196.443	n	chemotaxis	signal transduction response regulator, receiver domain	SpoE II output domain
	MSR1L_21200	?	MGMSRv2_2124	2.250.192	n	transporter	arsenical pump-driving ATPase	*arsA* ; operon arsenical resistance
	MSR1L_21370	2.280.101	MGMSRv2_2142	?	n	transporter	ABC-transporter, ATPase component	amno acid transport?
	MSR1L_21600	2.297.952	MGMSRv2_2163	?	n	transporter	TRAP-transporter, DctM	C4-dicarboxylate transport
	MSR1L_21620	2.301.213	MGMSRv2_2165	?	n	transporter	TRAP-transporter (DctM-DctQ fused)	C4-dicarboxylate transport
	MSR1L_21840	2.332.970	MGMSRv2_2186	?	n	cell division	DNA translocase FtK	
	MSR1L_22290	2.376.603	MGMSRv2_2232	?	n	?	DUF1285 protein	
	MSR1L_22710	2.420.191	MGMSRv2_2274	?	n	DNA	replicative helicase	
	MSR1L_06450	640.814	MGMSRv2_2462	2.576.698	n	?	protein of unknown function	1 TM (C-terminal)
	MSR1L_38450	4.016.350	MGMSRv2_2470	?	n	regulator	RNA polymerase sigma-70 factor RpoE	ECF family
	MSR1L_38410	4.013.407	MGMSRv2_2474	?	n	DNA	DNA mismatch repair protein MutL	
	MSR1L_38130	3.988.532	MGMSRv2_2501	?	n	cell division	cell division protein FtsQ	
	MSR1L_37510	3.931.308	MGMSRv2_2614	?	n	phage	bacteriophage capsid protein	
	MSR1L_36810	3.866.987	MGMSRv2_2680	?	n	regulator	RecX family protein	interaction with RecA, modulation of RecA activity
	MSR1L_36680	3.853.609	MGMSRv2_2693	?	n	redox	cyt c 551	
	MSR1L_36410	3.827.540	MGMSRv2_2719	2.820.417	n	transporter	ABC transporter, transmembrane region	
	MSR1L_36380	3.822.575	MGMSRv2_2722	?	n	cofactor biosynthesis	decarboxylase	*ubiD* ; ubiquinone biosynthesis

	MSR1L_36360	3.819.429	MGMSRv2_2724	2.829.888	n	chemotaxis	methyl-accepting chemotaxis protein	sCache_2
	MSR1L_35190	3.697.147	MGMSRv2_2838	?	n	signaling, His-kin	His-kinase	?
	MSR1L_34390	3.623.849	MGMSRv2_2917	3.016.900	n	cell cycle regulation?	histidine phosphotransferase	COG5385, downstream: *cheAWYBR* !
	MSR1L_34150	3.600.893	MGMSRv2_2954	?	n	redox	cyt c oxidase cbb3-type, subunit I	*fixN* /*ccoN* ; 12 TM
	MSR1L_33860	3.580.323	MGMSRv2_2984	3.071.684	n	flagellum	flagellar biosynthetic protein FliR	
	MSR1L_33760	3.567.138	MGMSRv2_2994	?	n	flagellum	flagellar hook length control protein FliK	
	MSR1L_33740	3.564.464	MGMSRv2_2996	3.087.857	n	flagellum	flagellar hook protein FlgE	
	MSR1L_33740	?	MGMSRv2_2996	3.087.850	n	flagellum	flagellar hook protein FlgE	
	MSR1L_33720	3.560.367	MGMSRv2_2998	?	n	redox	aldehyde dehydrogenase	
	MSR1L_33160	3.504.960	MGMSRv2_3027	3.120.125	n	celll wall	soluble lytic transglycosylase	murein degradation
	MSR1L_28770	3.049.447	MGMSRv2_3148	3.227.887	n	redox	cyt c-type protein, NapC/NirT multiheme cyt superfamily	Fur regulator right downstream (reverse 3149); Fur family protein upstream close (reverse 3137); NapC/NirT cytochrome c family
	MSR1L_29160	3.086.350	MGMSRv2_3189	?	n	redox	heme lyase CcmF	*ccmF*
	MSR1L_29180	3.088.874	MGMSRv2_3191	?	n	redox	cyt c-type biogenesis protein CcmH	*ccmH*
	MSR1L_29750	3.149.867	MGMSRv2_3248	?	n	cell wall	surface protein with repetitive structure	unique domain structure with Big_6 and Big_3_3
	MSR1L_29770	3.155.623	MGMSRv2_3251	?	n	transporter	RND efflux pump, membrane fusion protein	TiGR01730; 11 proteins in MSR-1
	MSR1L_29780	3.156.356	MGMSRv2_3252	?	n	transporter	biotin-lipoyl like domain of membrane fusion protein	
	MSR1L_30070	3.183.320	MGMSRv2_3280	3.358.689	n	RNA	RsmD	16S rRNA (guanine(966)-N(2))-methyltransferase, operon encoding RNA modifying proteins
	MSR1L_30080	3.183.944	MGMSRv2_3281	?	n	transporter?	Bax 1 inhibitor	7 TM; pH-dependent calcium leak across membrane
	MSR1L_30090	3.185.910	MGMSRv2_3282	?	n	transporter	ABC-transporter, substrate-binding protein	branched chain amino acids?
	MSR1L_30300	3.207.002	MGMSRv2_3303	3.381.111	n	(close to nitrogen)	conserved protein of unknown function	in same direction right downstream recombinase (3302); a little further *glnK* and *amtB* (3299, 3298)
	MSR1L_30380	3.212.388	MGMSRv2_3311	?	n	cell division	Maf-like protein	putative inhibitor of septum formation in euk, bacteria, archaea
	MSR1L_30390	?	MGMSRv2_3312	3.388.134	n	regulator, (close to cell division)	pyruvate, phosphate dikinase regulatory protein	in operon right upstream of maf (septum formation protein); heme biosyn genes downstream reverse
	MSR1L_30580	3.229.922	MGMSRv2_3331	?	n	signaling, His-kin	His-kinase	Chase, no HAMP, His-Kin

MSR1L_30940	?	MGMSRv2_3376	3.447.083	n	cell wall	NAD-dependent epimerase/dehydratase	
MSR1L_31930	3.377.375	MGMSRv2_3485	?	n	cofactor biosynthesis	GTP cyclohydrolase-2	*ribA* ; riboflavin biosynthesis; cofactor of reductases?
MSR1L_27290	2.903.192	MGMSRv2_3596	?	n	peptidase	ATP-dependent ClpA-ClpP serine protease, specificity subunit ClpA	
MSR1L_27410	2.918.802	MGMSRv2_3608	3.695.199	n	tRNA, (close to chemotaxis)	tRNA N(6)-(t(6)A) methylthiotransferase	MtaB (radical SAM)
MSR1L_27740	2.953.002	MGMSRv2_3644	3.731.153	n	signaling, His-kin	His-kinase	
MSR1L_27830	2.962.914	MGMSRv2_3653	3.740.796	n	RNA	ribosome-binding factor A	*rbfA*
MSR1L_28070	2.984.384	MGMSRv2_3680	?	n	transporter	multidrug resistance protein	*norM* ? 11 TM; 2 MatE domains
MSR1L_28090	2.986.821	MGMSRv2_3682	3.765.579	n	signaling	EAL-domain protein	PF00990; 6 proteins in MSR-1
MSR1L_27100	2.876.769	MGMSRv2_3696	?	n	nitrogen	glutamate synthase [NADPH], small chain	*gltD;* iron -sulfur cluster binding
MSR1L_27050	2.874.384	MGMSRv2_3702	3.785.117	n	oxygen	bacterioferritin - Bfr1	operon of 2 brf genes (deletion: no phenotype (R. Uebe pers. comm.)
MSR1L_26040	2.766.830	MGMSRv2_3810	3.904.094	n	signaling	sensor histidine kinase, x domain + PAS/PAC domain	N-terminal DUF3365
MSR1L_25790	2.738.640	MGMSRv2_3835	?	n	regulator	metal-sensitive transcriptional repressor	Cu, Ni, Co ion binding (Cys, His); transcriptional de-repression when metal bound
MSR1L_25430	2.702.740	MGMSRv2_3871	3.964.834	n	transporter	oligopeptide transporter subunit, periplasmic-binding component of ABC superfamily transporter	*oppA*
MSR1L_24980	2.658.206	MGMSRv2_3916	4.009.636	n	RNA	ATP-dependent RNA helicase SUV3, mitochondrial	
MSR1L_24760	2.638.710	MGMSRv2_3937	?	n	cofactor biosynthesis	aminotransferase	*pabB* ; para-aminobenzoate biosynthesis
MSR1L_24200	2.582.539	MGMSRv2_4000	?	n	metal-binding?	MEMO1 family protein	TIGR00296 + TIGR04335
MSR1L_24180	?	MGMSRv2_4002	4.089.064	n	cell wall	membrane lipid lysyl-group transfer protein	7 TM
MSR1L_24180	?	MGMSRv2_4002	4.089.287	n	cell wall	membrane lipid lysyl-group transfer protein	7 TM
MSR1L_23570	2.518.977	MGMSRv2_4044	4.141.463	n	?	conserved protein of unknown function	
MSR1L_23430/MSR1L_23440	2.506.037	MGMSRv2_4057	?	n	cell wall	surface antigen domain protein	*omp*
MSR1L_23090	?	MGMSRv2_4091	4.188.964	n	signaling, His-kin/RR	signal transduction response regulator, receiver domain + PAS + HisKA + EAL + GGDF	operon 4085-4092! starts with *fliI* , *divk* right downstream of 4091!
MSR1L_39290	4.098.592	MGMSRv2_4209	?	n	TPR	TPR-domain protein	glycosyl transferase family 9
MSR1L_39470	4.121.693	MGMSRv2_4227	4.331.590	n	metabolism	pyruvate, phosphate dikinase	*ppdK*

	Locus-tag MSR1(L), GenBank acc. CP027526, CP027527 (4)	Insertion position MSR1(L)	Locus-tag MGMSRv2, GenBank acc. HG794546 (5)	Insertion Position in MGMSRv2	inMAI = n, exMAI = y	(putative) functional category	Annotation	Comments
inMAI	**MSR1L_02710**	274.133	**MGMSRv2_2308**	2.443.699	y		**vWFA-protein**	
	MSR1L_02670	271.404	MGMSRv2_2312	2.446.524	y	iron	FeoB1	
	MSR1L_02670	271.169	MGMSRv2_2312	2.446.759	y	iron	FeoB1	
	MSR1L_02670	270.945	MGMSRv2_2312	2.447.282	y	iron	FeoB1	
	MSR1L_02670	270.908	MGMSRv2_2312	2.447.396	y	iron	FeoB1	
	MSR1L_02670	270.213	MGMSRv2_2312	2.447.564	y	iron	FeoB1	
	MSR1L_02660	?	MGMSRv2_2313	2.447.834	y	iron	FeoA1	
	**MSR1L_03790**	362.043	**MGMSRv2_2333**	?	y		**transposase**	
	**MSR1L_03720**	356.696	**MGMSRv2_2342**	?	y		**uncharacterized protein**	
	MSR1L_03490	?	MGMSRv2_2367	2.488.707	y		MamS	
	MSR1L_03490	?	MGMSRv2_2367	?	y		MamS	
	MSR1L_03470	?	MGMSRv2_2368	2.489.180	y		MamB	
	MSR1L_03450	?	MGMSRv2_2370	2.490.062	y		MamQ	
	MSR1L_03450	?	MGMSRv2_2370	2.490.322	y		MamQ	
	MSR1L_03440	?	MGMSRv2_2371	2.491.288	y		MamA	
	MSR1L_03440	?	MGMSRv2_2371	2.491.553	y		MamA	
	MSR1L_03430	?	MGMSRv2_2372	2.491.798	y	magnetochrome	MamP	
	MSR1L_03420	?	MGMSRv2_2373	2.492.513	y		MamO	
	MSR1L_03420	?	MGMSRv2_2373	2.492.691	y		MamO	
	MSR1L_03420	?	MGMSRv2_2373	2.492.725	y		MamO	
	MSR1L_03420	?	MGMSRv2_2373	2.493.657	y		MamO	
	MSR1L_03420	?	MGMSRv2_2373	2.493.676	y		MamO	
	MSR1L_03420	?	MGMSRv2_2373	2.494.252	y		MamO	
	MSR1L_03410	?	MGMSRv2_2374	2.494.949	y		MamN	
	MSR1L_03410	?	MGMSRv2_2374	2.495.482	y		MamN	
	MSR1L_03410	?	MGMSRv2_2374	2.495.507	y		MamN	
	MSR1L_03400	?	MGMSRv2_2375	2.495.711	y		MamM	
	MSR1L_03400	?	MGMSRv2_2375	2.496.524	y		MamM	
	MSR1L_03400	?	MGMSRv2_2375	2.496.588	y		MamM	
	MSR1L_03390	327.647	MGMSRv2_2376	2.496.966	y		MamL	
	MSR1L_03380	327.528	MGMSRv2_2377	?	y		MamK	
	MSR1L_03380	?	MGMSRv2_2378	2.498.200	y		MamJ	
	MSR1L_03380	?	MGMSRv2_2378	2.498.495	y		MamJ	
	MSR1L_03380	?	MGMSRv2_2378	2.498.950	y		MamJ	
	MSR1L_03360	?	MGMSRv2_2379	2.500.164	y	magnetochrome	MamE	
	MSR1L_03360	?	MGMSRv2_2379	2.500.909	y	magnetochrome	MamE	
	MSR1L_03360	?	MGMSRv2_2379	2.501.121	y	magnetochrome	MamE	
	MSR1L_03360	?	MGMSRv2_2379	2.501.231	y	magnetochrome	MamE	
	MSR1L_03360	?	MGMSRv2_2379	2.501.283	y	magnetochrome	MamE	
	MSR1L_03360	?	MGMSRv2_2379	2.501.362	y	magnetochrome	MamE	
	MSR1L_03360	?	MGMSRv2_2379	2.501.601	y	magnetochrome	MamE	
	MSR1L_03360	?	MGMSRv2_2379	2.501.700	y	magnetochrome	MamE	

	MSR1L_03350	?	MGMSRv2_2380	2.501.812	y		MamI	
	MSR1L_03350	?	MGMSRv2_2380	2.501.906	y		MamI	
	MSR1L_03340	?	MGMSRv2_2381	2.502.087	y		MamH	
	MSR1L_03340	?	MGMSRv2_2381	2.502.815	y		MamH	
	MSR1L_03340	?	MGMSRv2_2381	2.503.101	y		MamH	
	MSR1L_03180	308.340	MGMSRv2_2396	2.515.320	y		Mms6	
