# Supplementary material for: Genome-Wide Identification of Essential and Auxiliary Gene Sets for Magnetosome Biosynthesis in Magnetospirillum gryphiswaldense
Source: mSystems. 2020 Nov 17;5(6):e00565-20. doi: 10.1128/mSystems.00565-20 (PMC7676999; doi:10.1128/mSystems.00565-20)
Supplement: FIG S4 [file mSystems.00565-20-sf004.pdf]

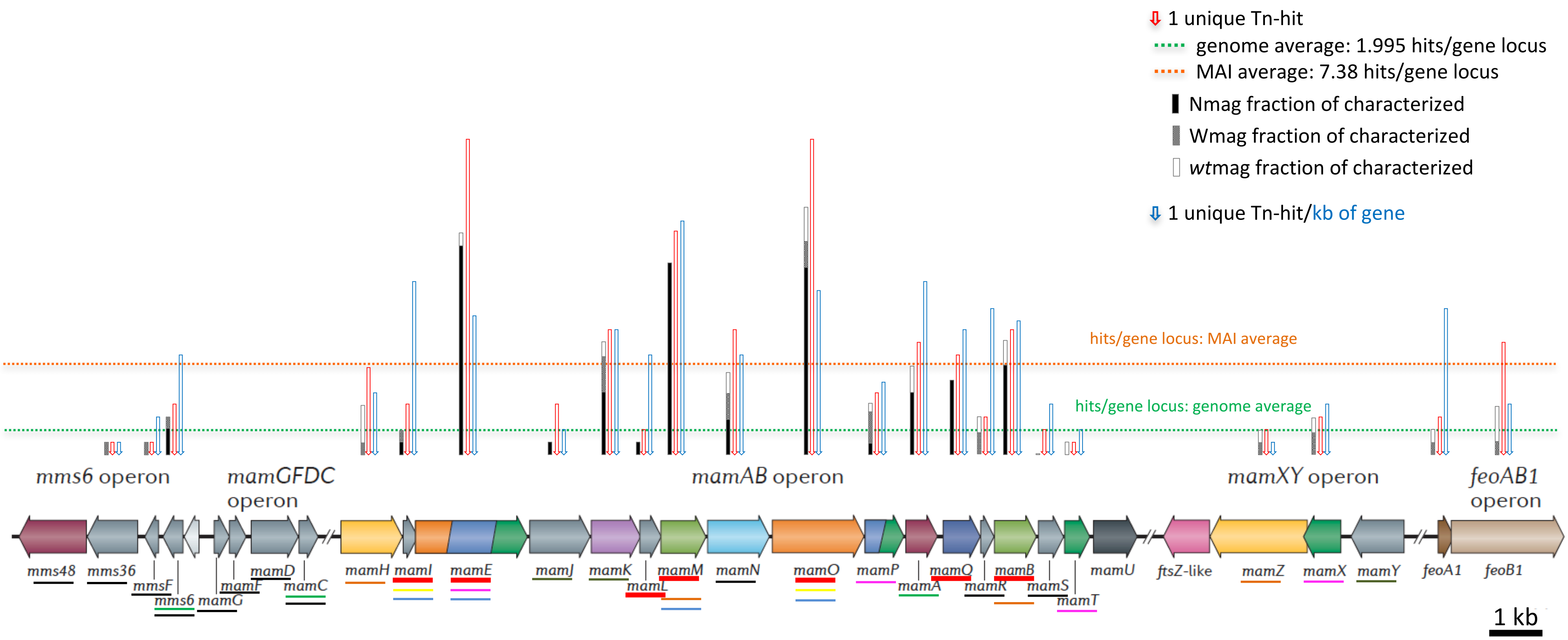
1 unique Tn-hit
genome average: 1.995 hits/gene locus
MAI average: 7.38 hits/gene locus
Nmag fraction of characterized
Wmag fraction of characterized
wtmag fraction of characterized
1 unique Tn-hit/kb of gene
hits/gene locus: MAI average
hits/gene locus: genome average
mms6 operon
mamGFDC operon
mamAB operon
mamXY operon
feoAB1 operon
mms48
mms36
mmsF
mms6
mamG
mamF
mamD
mamC
mamH
mamI
mamE
mamJ
mamK
mamL
mamM
mamN
mamO
mamP
mamA
mamQ
mamR
mamB
mamS
mamT
mamU
ftsZ-like
mamZ
mamX
mamY
feoA1
feoB1
1 kb
