# Supplementary material for: Genome-Wide Identification of Essential and Auxiliary Gene Sets for Magnetosome Biosynthesis in Magnetospirillum gryphiswaldense
Source: mSystems. 2020 Nov 17;5(6):e00565-20. doi: 10.1128/mSystems.00565-20 (PMC7676999; doi:10.1128/mSystems.00565-20)
Supplement: TABLE S3 [file mSystems.00565-20-st003.pdf]

Table S3. exMAI N/Wmag genes associated with overrepresented GO-terms identified by FET-analysis against the genomic background

	MGMSRv2_	MSR1L_	GO-terms	Magnetic phenotype of Tn5 insertion alleles (nc: not-characterized beyond colony color)
1	1252	10050	4-coumarate-CoA ligase activity	Wmag
2	1722	5930	calcium ion binding	Wmag
3	266	19360	calcium ion binding-external encapsulating structure	Wmag+nc
4	1279	9800	dimethylallyltranstransferase activity-geranyltranstransferase activity	Wmag
5	1417	8250	external encapsulating structure	Wmag
6	1082	11790	guanosine tetraphosphate metabolic process-diphosphoric monoester hydrolase activity-GTP diphosphokinase activity-guanosine-3',5'-bis(diphosphate) 3'-diphosphatase activity-purine ribonucleoside bisphosphate metabolic process	Wmag+WTmag
7	398	18150	guanosine-5'-triphosphate,3'-diphosphate diphosphatase activity-exopolyphosphatase activity	Wmag
8	3560	32700	homocysteine biosynthetic process-homocysteine metabolic process	Wmag
9	3966	24500	hydroxyacylglutathione hydrolase activity	Wmag
10	2673	36930	methylmalonyl-CoA mutase activity-tetrapyrrole binding-cobalamin binding	Wmag
11	3255	29810	methylmalonyl-CoA mutase activity-tetrapyrrole binding-cobalamin binding	Wmag
12	2474	38410	mismatch repair-mismatched DNA binding	Wmag+nc
13	450	17640	oxidation-reduction process	Wmag
14	1295	9720	oxidation-reduction process	Wmag
15	1555	14910	oxidation-reduction process	Wmag
16	2011	2270	oxidation-reduction process	Wmag
17	2273	22700	oxidation-reduction process	6Wmag+WTmag
18	2950	34190	oxidation-reduction process	Wmag
19	3942	24710	oxidation-reduction process	Wmag
20	853	14090	oxidation-reduction process-**alpha-1,4-glucan synthase** activity-starch synthase activity-glycogen (starch) synthase activity-UDP-glucosyltransferase activity-glucosyltransferase activity	Wmag
21	1617	15530	oxidation-reduction process-**glutaryl-CoA dehydrogenase** activity	Wmag
22	1403	8390	oxidation-reduction process-oxidoreductase activity, acting on other nitrogenous compounds as donors, cytochrome as acceptor-tetrapyrrole binding-**hydroxylamine reductase** activity-nitrite reductase (NO-forming) activity	Wmag
23	1716	5990	oxidation-reduction process-oxidoreductase activity, acting on other nitrogenous compounds as donors, cytochrome as acceptor-tetrapyrrole binding-**nitric oxide reductase** activity	2 Wmag+nc
24	469	17440	oxidation-reduction process-**phosphoadenylyl-sulfate reductase** (thioredoxin) activity-sulfate assimilation, phosphoadenylyl sulfate reduction by phosphoadenylyl-sulfate reductase (thioredoxin)-oxidoreductase activity, acting on a sulfur group of donors, disulfide as acceptor	Wmag+WTmag+2nc
25	470	17430	oxidation-reduction process-**sulfate adenylyltransferase** activity-sulfate adenylyltransferase (ATP) activity-sulfate reduction	Wmag+WTmag+2nc
26	134	450	protein histidine kinase activity-phosphorelay sensor kinase activity- phosphotransferase activity, nitrogenous group as acceptor-protein kinase activity	Wmag
27	1113	11550	protein histidine kinase activity-phosphorelay sensor kinase activity- phosphotransferase activity, nitrogenous group as acceptor-protein kinase activity	Wmag+nc
28	732	16320	protein histidine kinase activity-phosphorelay sensor kinase activity- phosphotransferase activity, nitrogenous group as acceptor-protein kinase activity	Wmag
29	1231	10260	protein histidine kinase activity-phosphorelay sensor kinase activity- phosphotransferase activity, nitrogenous group as acceptor-protein kinase activity	Wmag+nc
30	1777	5370	protein histidine kinase activity-phosphorelay sensor kinase activity- phosphotransferase activity, nitrogenous group as acceptor-protein kinase activity	Wmag
31	4151	38770	protein histidine kinase activity-phosphorelay sensor kinase activity- phosphotransferase activity, nitrogenous group as acceptor-protein kinase activity	Wmag
32	2976	33940	secretion-secretion by cell-peptide secretion-protein secretion	Wmag
33	3625	27560	secretion-secretion by cell-peptide secretion-protein secretion	Wmag
34	3736	26700	sirohydrochlorin cobaltochelatase activity	Wmag
35	3376	30940	UDP-glucuronate decarboxylase activity	Wmag+5 WTmag+nc
36	2540	37510	virion assembly	Wmag
