# Supplementary material for: Genome-Wide Identification of Essential and Auxiliary Gene Sets for Magnetosome Biosynthesis in Magnetospirillum gryphiswaldense
Source: mSystems. 2020 Nov 17;5(6):e00565-20. doi: 10.1128/mSystems.00565-20 (PMC7676999; doi:10.1128/mSystems.00565-20)
Supplement: TABLE S4 [file mSystems.00565-20-st004.pdf]

Table S4. Characteristics of Mgryph deletion mutants* in genes for which Tn5-insertion yielded a magnetosome phenotype

**Locus-tag MSR1(L)_**	**Locus-tag MGMSRv2_**	**Gene name**	**Magnetic phenotype (Δ) compared to WT**	**TEM (Δ)**
29190	3192	***ccmI* (*cycH)***	less magnetosomes	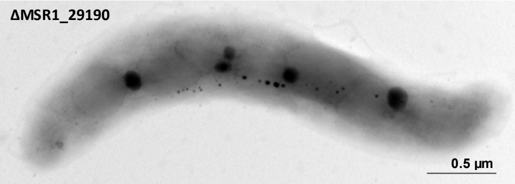
22700	2273	***dsbA***	shorter chains	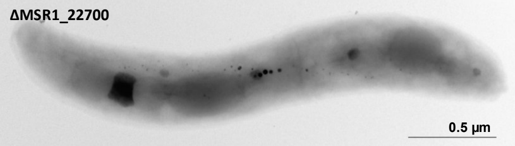
17040	511	***dsbB***	smaller and less magnetosomes	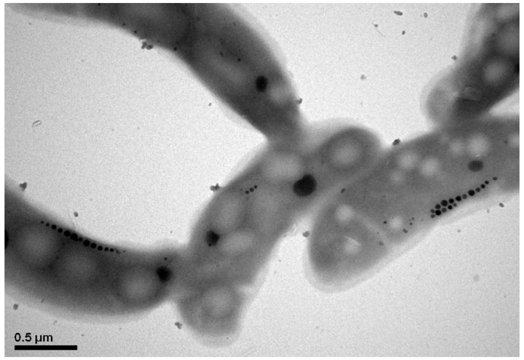
17450-17420	468-471	***sir-cysH-cysD-cysC***	significantly increased number of defective crystals, higher prevalence of cells with double magnetosome chains	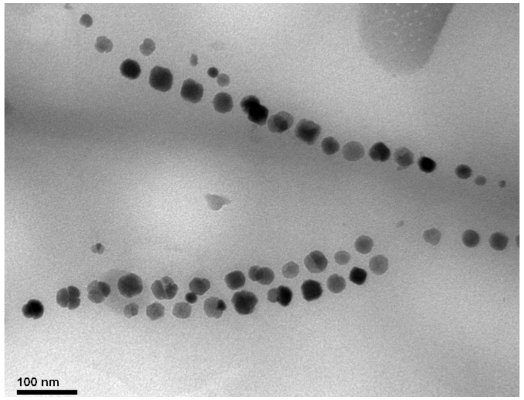

*grown microaerobically in flask standard medium (FSM, U. Heyen and D. Schüler, Appl Microbiol Biotechnol 61, 2003, doi: 10.1007/s00253-002-1219-x)
