# Supplementary material for: Genome-Wide Identification of Essential and Auxiliary Gene Sets for Magnetosome Biosynthesis in Magnetospirillum gryphiswaldense
Source: mSystems. 2020 Nov 17;5(6):e00565-20. doi: 10.1128/mSystems.00565-20 (PMC7676999; doi:10.1128/mSystems.00565-20)
Supplement: TABLE S1 [file mSystems.00565-20-st001.pdf]

Table S1 A. Bacterial strains and plasmids used in this study.

Bacterial strains / plasmids	Relevant characteristics	Reference
**Strains**		
M. gryphiswaldense		
WT		D. Schüler and M. Köhler, Zentralblatt Mikrobiol 147, 1992
E. coli		
DH5α	F$^-$ *sup*E44 Δ*lac*U169 (Φ80 *lacZ*DM15) *hsd*R17 *recA*1 *end*A1 *gyr*A96 *thi*-1 *rel*A1	Invitrogen
WM3064	donor strain for conjugation, *dap* auxotroph (*thr*B1004 *pro thi rps*L *hsd*S *lac*ZΔM15 RP4-1360 Δ(*ara*BAD)567 Δ*dap*A1341::[erm pir])	W. Metcalf (unpublished)
**Plasmids**		
pBAM1 (original)		E. Martínez-García *et al.*, BMC Microbiol 11, 2011
GeneArt® plasmid	synthetic transposase ($P_{mamDC45}$)	This study
pBAMOpt	synthetic transposase (P_{mamDC})	This study

Table S1 B. Primers used in this study.

Name	Sequence (5'-3')
IB056	ATGGAACCTGGCAGATCAGAAGT
IB057	TCAAAGAACAATCCAGAACTCTTGG
IB058	ATGAGGAAGAGCGGTTGCGC
IB059	TCATCCTGCGAGAACGGCGA
IB060	ATGATTGAAATTGGCGAGACCA
IB061	CTCAATGAGACCTTCTACATCGACTG
IB062	ATGGCAGTAAGCGATGCGG
IB063	TCACTGCACGGTCATCCACA
IB064	ATGGGTACGCCAGGGGG
IB065	TTATTTCGGAACCAGTATGGAAAGC
IB140	ATGGAACCTGGCAGATCAGA
IB141	CCACATCACCATTGAACATG
ARB6	GGCACGCGTCGACTAGTACNNNNNNNNNNACGCC
ARB2	GGCACGCGTCGACTAGTAC
Me-O-extF	CGGTTTACAAGCATAACTAGTGCGGC
Me-O-intF	AGAGGATCCCCGGGTACCGAGCTCG
Me-I-extR	CTCGTTTCACGCTGAATATGGCTC
Me-I-intR	CAGTTTTATTGTTCATGATGATATA
